# London Trauma Conference December 2022: Abstracts

**DOI:** 10.1186/s13049-023-01100-1

**Published:** 2023-10-24

**Oral presentations**

## O1 Predicting opportunities for improvement in trauma care using machine learning

### Jonatan Attergrim^1,*^, Kelvin Szolnoky^2^, Lovisa Strömmer^3^, Olof Brattström^4^, Martin Jacobsson^5^, Martin Gerdin Wärnberg^1^

#### ^1^Department of Global Public Health, Karolinska Institutet, Stockholm, Sweden. ^2^Department of Medical Epidemiology and Biostatistics Karolinska Institutet, Stockholm, Sweden. ^3^Department of Clinical Science, Intervention and Technology, Division of Surgery, Karolinska Institute, Karolinska University Hospital Huddinge, 14186 Stockholm, Sweden. ^4^Perioperative Medicine and Intensive Care, Karolinska University Hospital, Solna, Stockholm, Sweden. ^5^Department of Biomedical Engineering and Health Systems, KTH Royal Institute of Technology, Huddinge, Sweden

##### *Correspondence: Jonatan.attergrim@ki.se

***Scandinavian Journal of Trauma, Resuscitation and Emergency Medicine*** 2023, **31(1)**: O1

**Background: **Multidisciplinary mortality and morbidity review (MoM) is the core of programs that aim to improve the quality of trauma care and is used to identify and address opportunities for improvement (OFI) [1]. Case selection for MoM relies heavily on audit filters, a process associated with high frequencies of false positives. The use of trauma injury scores has been proposed as an alternative, but performance is also poor [2]. Our aim was to develop, validate and compare the performance of different machine learning (ML) based models for predicting opportunities for improvement.

**Methods: **We included all trauma patients from the Karolinska University Hospital trauma registry and trauma care quality database assessed between 2014 and 2021. OFI was defined as a binary variable representing a consensus decision from the Mortality and Morbidity Conference regarding the presence of at least one OFI. The data was split into a training (80%) and test set (20%). We developed seven ML models with 45 predictors and compared the performance between models. We also compared the performance of the models with currently used audit filters. Performance was measured using AUC, accuracy and Integrated Calibration Index. A resampling approach was used to estimate confidence intervals.

**Results: **We included 6313 patients where OFI were present in 431 (6.83%) patients. The currently used audit filters (AUC: 0. 0.627, accuracy: 0.356) was outperformed by all developed models. Our best performing model was random forest (AUC: 0.794, accuracy: 0.933, ICI: 0.028) followed by light gradient-boosting machine (AUC: 0.790, accuracy: 0.932, ICI: 0.037).

**Conclusions: **Machine learning models outperform audit filters based on established quality indicators and could prove to be valuable additions in the screening for OFI. Further research is needed on how to further increase performance as well as how to optimally apply developed models into trauma quality programs.

**References**

1. World Health Organization. Guidelines for trauma quality improvement programmes [Internet]. World Health Organization; 2009. Available from: http://whqlibdoc.who.int/publications/2009/9789241597746_eng.pdf?ua=1
2. Heim C, Cole E, West A, Tai N, Brohi K. Survival prediction algorithms miss significant opportunities for improvement if used for case selection in trauma quality improvement programs. Injury. 2016 Sep;47(9):1960–5.

## O2 Prehospital resuscitative thoracotomy for traumatic cardiac arrest: outcomes of 601 cases at London’s air ambulance

### RM Greenhalgh^1,2,*^, S Aziz^1,2^, A Whitehouse^1^, S Read^1^, E Foster^2^, C.L. Henry^1^, M Marsden^3,4^, R Lendrum^1,2^, D Lockey^1,2,3^, M Christian^1^, Z.B. Perkins^1,2^

#### ^1^London’s Air Ambulance Charity, London, UK. ^2^The Royal London Hospital, Barts Health NHS Trust, London UK. ^3^Centre for Trauma Sciences, Queen Mary University of London, UK. ^4^Academic Department of Military Surgery and Trauma, Royal Centre for Defence Medicine, Birmingham, UK

##### *Correspondence: Robert.Greenhalgh3@nhs.net

***Scandinavian Journal of Trauma, Resuscitation and Emergency Medicine*** 2023, **31(1)**: O2

**Background: **Resuscitative thoracotomy (RT) is an established treatment for patients in traumatic cardiac arrest (TCA). However, the indications to perform the procedure in the pre-hospital setting remain unclear.

**Methods: **A retrospective observational study of all patients who underwent pre-hospital RT by London’s Air Ambulance between January 1999 and December 2019. Outcomes were survived event (return of spontaneous circulation [ROSC] sustained until hospital admission), survived to hospital discharge, and survived with favourable neurological outcome (Cerebral Performance Category (CPC) score of ≥ 2).

**Results: **During the 20-year study period, 601 patients underwent prehospital RT. Median age was 25 (range: 2–98) years, 90% were male, and 88% suffered a penetrating mechanism of injury. The median time between emergency call and onset of TCA was 13 (6 to 24) minutes, and between emergency call and RT was 22 (17–30) minutes. The cause of TCA was exsanguination (69.8%), cardiac tamponade (17.8%), both pathologies (12.1%), or other/unclear (1.0%). Significantly more patients with cardiac tamponade survived the event (55.7% vs. 24.9%, OR 4.5 (2.9 to 7.2), *p* < 0.0001) and survived to hospital discharge (20.8% versus 1.9%, OR 13.4 (5.9 to 29.1), *p* < 0.0001) when compared to exsanguinated patients. The proportion of survivors with favourable neurological outcome was similar between groups (72.7% versus 87.5%, *p* = 0.638). There were no survivors beyond 15 min of TCA for Cardiac Tamponade and none beyond 5 min of TCA following exsanguination.

**Conclusions: **Pre-hospital RT is indicated for all patients in TCA where the cause might be Cardiac Tamponade and the duration has been less than 15 min. Outcomes following RT for TCA caused by exsanguination are poor, and novel approaches to resuscitating this group of patients are required.

## O3 Six years of hospital-based ECPR for out-of-hospital cardiac arrest in Helsinki: an observational cohort study

### Tuukka Puolakka^1,*^, Ari Salo^1^, Marjut Varpula^2^, Jouni Nurmi^1^, Markus Skrifvars^1^, Erika Wilkman^3^, Karl Lemström^4^, Markku Kuisma^1^

#### ^1^Department of Emergency Medicine and Services, Helsinki University Hospital and the University of Helsinki, Helsinki, Finland. ^2^Department of Cardiology, Helsinki University Hospital and the University of Helsinki, Helsinki, Finland. ^3^Department of Anaesthesia and Intensive Care Medicine, Helsinki University Hospital and the University of Helsinki, Helsinki, Finland. ^4^Department of Cardiac Surgery, Helsinki University Hospital and the University of Helsinki, Helsinki, Finland

##### *Correspondence: tuukka.puolakka@hus.fi

***Scandinavian Journal of Trauma, Resuscitation and Emergency Medicine*** 2023, **31(1)**: O3

**Background: **Extracorporeal cardiopulmonary resuscitation (ECPR) is an emerging treatment for refractory cardiac arrest requiring a complex and time-critical chain of care [1–3]. The aim of this study was to report the real-life performance of a single-center hospital-based ECPR-protocol for refractory out-of-hospital cardiac arrest (OHCA).

**Materials and methods: **All cases of OHCA between 1.1.2016 and 31.12.2021 where the prehospital physician activated the ECPR protocol in the Helsinki University Hospital catchment area were included in the study.

**Results: **The ECPR protocol was activated in 73 cases of normothermic OHCA. The mean (standard deviation) patient age was 54.4 (11.3) years and 67 (91.8%) of them were male. The arrest was witnessed in 67 (91.8%) and initial rhythm was shockable in 61 (83.6%) cases. In 10 (13.7%) cases a preceding ECG meeting the criteria for ST-elevation myocardial infarction was registered before arrest. All patients received mechanical CPR, adrenaline, and amiodarone. Seventy (95.9%) patients were endotracheally intubated. The median (interquartile range, IQR) highest prehospital etCO2 was 5.5 (4.0–6.9) kPa. The median (IQR) ambulance response time was 8.5 (6.8–11.1), mechanical CPR initiation time 10.8 (6.2–19.0) and arrest-to-door time 56.3 (45.2–68.1) minutes. A total of 37 (50.7%) patients were connected to extracorporeal membrane oxygenation (ECMO) in 23 (16.3–30.2) minutes with a total arrest-to-ECMO time of 84.4 (71.1–105.2) minutes. In the ECMO-group, 13 (35.1%) patients survived to discharge with 12 (32.4%) patients alive after one year and 11 (29.7%) with a Cerebral Performance Category 1–2. In the non-ECMO group six patients (16.7%) received permanent ROSC immediately after hospital arrival. All of them survived to discharge with a CPC 1–2.

**Conclusions: **Only half of the patients with OHCA who met the ECPR-criteria eventually received ECPR in the hospital. Every fourth patient who met the criteria and every third patient who received ECMO treatment survived with a good outcome.

**References**

1. Soar J, Böttiger BW, Carli P, Couper K, Deakin CD, Djärv T, Lott C, Olasveengen T, Paal P, Pellis T, Perkins GD, Sandroni C, Nolan JP. European Resuscitation Council Guidelines 2021: Adult advanced life support. Resuscitation. 2021;161:115–151.
2. Yannopoulos D, Bartos J, Raveendran G, Walser E, Connett J, Murray TA, Collins G, Zhang L, Kalra R, Kosmopoulos M, John R, Shaffer A, Frascone RJ, Wesley K, Conterato M, Biros M, Tolar J, Aufderheide TP. Advanced reperfusion strategies for patients with out-of-hospital cardiac arrest and refractory ventricular fibrillation (ARREST): a phase 2, single centre, open-label, randomised controlled trial. Lancet. 2020;396:1807–1816.
3. Belohlavek J, Smalcova J, Rob D, Franek O, Smid O, Pokorna M, Horák J, Mrazek V, Kovarnik T, Zemanek D, Kral A, Havranek S, Kavalkova P, Kompelentova L, Tomková H, Mejstrik A, Valasek J, Peran D, Pekara J, Rulisek J, Balik M, Huptych M, Jarkovsky J, Malik J, Valerianova A, Mlejnsky F, Kolouch P, Havrankova P, Romportl D, Komarek A, Linhart A; Prague OHCA Study Group. Effect of Intra-arrest Transport, Extracorporeal Cardiopulmonary Resuscitation, and Immediate Invasive Assessment and Treatment on Functional Neurologic Outcome in Refractory Out-of-Hospital Cardiac Arrest: A Randomized Clinical Trial. JAMA 2022;327:737–747.

## O4 ThoraTed: a DIY Thoracotomy model providing an accessible, cost-efficient, reproducible, environmentally friendly solution for practising a low-incidence, time critical procedure

### Irini Yanny^1,*^, Tim Coats^1^

#### ^1^University Hospitals of Leicester, Leicester, UK

##### *Correspondence: iriniyanny@gmail.com

***Scandinavian Journal of Trauma, Resuscitation and Emergency Medicine*** 2023, **31(1)**: O4

**Background: **Emergency Physicians must be competent in thoracotomy. This is difficult as it is a high acuity, low incidence procedure. Teaching the decision-making principles can be effectively achieved via simulation. Gaining the psychomotor skill however is challenging and a lack of experience may affect cognitive biases when making decisions. There are courses but they are few and infrequent. Furthermore, whilst searching for a model for teaching, it was difficult to find something suitable. Cost precluded the hire of a high-fidelity Sim-Man (£5000 daily). Access to cadavers is difficult and expensive. Thus, ThoraTed came to life.

**Methods: **ThoraTed has all the layers required to perform a clamshell thoracotomy. He is made from readily available internet, hospital-based and household equipment. He can be re-set in between scenarios and re-used. After positive feedback at local teaching, ThoraTed went on tour to be tested in different settings, amongst Pre-hospital providers and Emergency Physicians. Feedback questionnaires were distributed post-session.

**Results: **There were 51 participants, 72.5% responded: 27% Pre-Hospital Care background and 73% Emergency Medicine. 78.4% had no thoracotomy experience. 35% had never seen one or attended a course. 100% of respondents agreed ThoraTed was useful to learn or revise the psychomotor skill. 94.6% agreed that ThoraTed improved their confidence in performing thoracotomy. The remainder were neutral. 96% of respondents would recommend ThoraTed as a training model.

**Conclusions: **Results reflected that even amongst those with previous thoracotomy experience, ThoraTed seemed an effective training aid, whilst remaining cost-effective and environmentally friendly. He is not a replacement for important cadaveric practice but provides a complimentary role, bridging the accessibility gap. He makes a useful addition to everyone’s thoracotomy teaching toolkit, helping providers at all levels of experience to either learn or revise the psychomotor skill and increase confidence in performing thoracotomy.

## O5 Critical decision analysis of pre-hospital major haemorrhage protocol activation

### Jared M Wohlgemut^1,*^, Heather McMullen^2^, Max Marsden^1^, Erhan Pisirir^3^, Rebecca S Stoner^1^, Evangelia Kyrimi^3^, William Marsh^3^, Zane B. Perkins^4^, Nigel R. M. Tai^5^

#### ^1^Centre for Trauma Sciences, Blizard Institute, Queen Mary University of London, London, UK. ^2^Global Public Health Unit, Centre for Public Health and Policy, Wolfson Institute of Population Health, Queen Mary University of London, London, UK. ^3^Department of Electrical Engineering and Computer Science, Queen Mary University of London, London, UK. ^4^London’s Air Ambulance, 17 th Floor, Royal London Hospital, London, UK. ^5^Trauma Service, Royal London Hospital, Barts Health NHS Trust, London, UK

##### *Correspondence: j.wohlgemut@qmul.ac.uk

***Scandinavian Journal of Trauma, Resuscitation and Emergency Medicine*** 2023, **31(1)**: O5

**Background: **Major haemorrhage protocol activation (MHPA) is designed to treat major bleeding by facilitating immediate blood product availability and institutional capacity readiness. In the London trauma system, two thirds of MHPA decisions occur pre-hospital. Understanding what influences the pre-hospital MHPA decision is a prerequisite to improving clinician decision-making processes. Our aim was to identify the critical determinants of the pre-hospital MHPA decision.

**Methods: **This prospective qualitative study was approved by the Health Research Authority (REC 22/PR/0089). Physicians from one helicopter emergency medical service (HEMS) were purposively sampled, and written informed consent was obtained. Cases where the MHPA decision was difficult within the last six months were included. Semi-structured interviews were conducted on the Microsoft Teams platform, according to the critical decision analysis method [1]. Video- and audio-recordings were transcribed and analysed according to the 6-step inductive thematic analysis method using NVivo software [2].

**Results: **Twelve interviews were conducted with nine participants: median age 37, median post-graduate experience 12 years (inter-quartile range (IQR) 10.5–14.8), and median HEMS experience 1 year (IQR 0.5–4.9). Cues for the MHPA decision included: hard evidence of bleeding, initial information fit with their mental model of a bleeding patient, patient deterioration, acknowledgement that injuries and bleeding can be missed, and when alternative diagnoses were considered less likely. Cues against the MHPA decision included: initial information did not fit with their mental model of a bleeding patient, responsive to resuscitation, uncertainty of clinical findings, bleeding judged to be non-severe, and when alternative diagnosis were considered more likely. When considering the MHPA decision, clinicians were influenced by previous experience, colleagues, time pressure, overwhelming task volume, and the desire to prevent delay while ensuring diagnostic accuracy and professional reputation.

**Conclusion: **There are multiple determinants of the pre-hospital MHPA decision. Acknowledging these cognitive processes will inform efforts to improve decision-making in trauma care.

**References**

1. Klein GC, Macgregor D. Critical Decision Method for Eliciting Knowledge. IEEE Transactions on Systems, Man and Cybernetics. 1989; 19(3): 462–72.
2. Braun V, Clarke V. Using thematic analysis in psychology. Qual Res Psychol. 2006; 3(2):77–101.

## O6 Pre-charging the defibrillator before rhythm analysis reduces hands-off time in patients with out-of-hospital cardiac arrest with shockable rhythm

### Bo Nees Iversen^1^, Carsten Meilandt^1,*^, Ulla Væggemose^1^, Christian J. Terkelsen^2^, Hans Kirkegaard^3^, Jesper Fjølner^1^

#### ^1^Prehospital Emergency Medical Services, Central Denmark Region, Aarhus, Denmark. ^2^Department of Cardiology, Aarhus University Hospital, Aarhus, Denmark. ^3^Research Centre for Emergency Medicine, Aarhus University Hospital, Aarhus, Denmark

##### *Correspondence: carmei@rm.dk

***Scandinavian Journal of Trauma, Resuscitation and Emergency Medicine*** 2023, **31(1)**: O6

**Background: **Minimising pauses during cardiopulmonary resuscitation (CPR) is a cardinal element in recent CPR guidelines [1]. Charging the defibrillator before analysing the rhythm during cardiac arrest introduces a combined rhythm analysis- and shock pause, potentially further minimising compression pauses. This “Precharge” method has mostly been investigated in manikin studies and real-world data is scarce [2].

**Method: **This cohort study included cardiac arrest patients in Central Denmark Region from January 2018 until March 2019. Adult patients subjected to at least one defibrillation were included. Patients with insufficient or uninterpretable transthoracic impedance data were excluded. Data were extracted from the Danish Cardiac Arrest Registry, the regional defibrillator database and patient records. The Precharge defibrillation method was implemented in the Emergency Medical Service in June 2018 and was mandatory after a training period of one month. All defibrillations were categorised according to their type: "Precharge" method, “Standard” method (separate analysis and shock pause with chest compressions during charging) and “Old” method (one pause for rhythm analysis, charging and shock).

**Results: **Impedance and outcome data were available for 178 of 287 patients and 523 defibrillation procedures were analysed. The Precharge method was associated with shorter median hands-off time per defibrillation procedure (7.6 (IQR 5.8–9.9) vs. 12.6 (IQR 10.0–16.4) seconds, *p* < 0.001) but longer pre-shock pause (4.0 (IQR 2.7–6.1) vs 1.7 (IQR 1.2–3.0) seconds, *p* < 0.001) when compared to Standard method. No increase in shocks to non-shockable rhythms or personnel was registered. Patients receiving only Precharge defibrillations had an increased adjusted odds ratio (aOR) for return of spontaneous circulation (aOR 2.91; 95%CI 1.09–7.8, *p* = 0.03).

**Conclusion: **Pre-charging the defibrillator reduced hands-off time during defibrillation procedures, reduces the total hands-off fraction and may be associated with increased return of spontaneous circulation in out-of-hospital cardiac arrest with shockable rhythm.

**References**

1. Soar J, Böttiger BW, Carli P, Couper K, Deakin CD, Djärv T, et al. European Resuscitation Council Guidelines 2021: Adult advanced life support. Resuscitation. 2021: 161:115–51.
2. Otto Q, Musiol S, Deakin CD, Morley P, Soar J. Anticipatory manual defibrillator charging during advanced life support: A scoping review. Resuscitation Plus. 2020: 1–2:100,004.

## O7 The effect of calcium chloride on hypocalcaemia when incorporated into pre-hospital blood transfusion protocols in major trauma patients

### Jennifer Scallan^1,*^, Joanne Griggs^2^, Russell Gritton^3^, Rachel Hawes^4^, Elaine Cole^5^, Ross Davenport.^6^

#### ^1^Queen Elizabeth Hospital, Birmingham, UK. ^2^Kent Surry Sussex Air Ambulance Service, UK. ^3^Royal London Hospital, Barts Health, UK. ^4^Great North Air Ambulance Service, UK. ^5,6^Centre for Trauma Sciences, Blizard Institute QMUL, UK

##### *Correspondence: Jennifer.Scallan@uhb.nhs.uk

***Scandinavian Journal of Trauma, Resuscitation and Emergency Medicine*** 2023, **31(1)**: O7

**Background: **Hypocalcaemia is associated with coagulopathy, hypotension and increased mortality in trauma patients [1]. Calcium is chelated by citrate in transfused blood components, a cofactor of multiple clotting factors and required for fibrin formation [2]. This study aimed to identify if the addition of calcium chloride (CaCl) to pre-hospital blood component transfusion protocols reduced the incidence of hypocalcaemia (ionised calcium (iCa) ≤ 1.00 mmol/L).

**Methods: **A retrospective observational study was performed on patients receiving pre-hospital blood components from four UK services in 2017–2021. Two of these services administered CaCl as part of their transfusion protocol. The iCa on arrival in the Emergency Department was compared between groups receiving and not receiving CaCl. Secondary outcomes included 24-h mortality and the effect of CaCl dose.

**Results: **265 patients were included, 78 did not receive CaCl and 156 did (81 received 5 ml 10% CaCl, 75 received 10 ml 10% CaCl). Hypocalcaemia incidence in the no CaCl group was significantly higher; 37% versus 8% (*p* < 0.0001, 95% CI 0.07–0.31). Emergency Department iCa levels were higher in the group receiving CaCl (1.22 mmol/L vs 1.070 mmol/L, 95% CI 0.13–0.21, *p* < 0.0001). There was no difference between either group in 24-h mortality (23% vs 22%, 95% CI 0.56–2.02, *p* = 0.8681). Variables associated with hypocalcaemia were total number of blood components administered (*p* = 0.0252), and ISS (*p* = 0.0196). For the no CaCl group, total blood products administered was inversely related to iCa (R^2^ 0.09, *p* = 0.005), median iCa following 2-unit transfusion was 1.02 mmol/L. Hypocalcaemia incidence in the 5 ml 10% CaCl and 10 ml 10% CaCl sub-groups were similar (11% vs 6%, 95% CI 0.20–1.76, *p* = 0.407).

**Conclusion: **CaCl administration in pre-hospital blood transfusion protocols is associated with a reduced incidence of hypocalcaemia. iCa reduces in a dose-dependent manner with blood component administration, therefore services could consider the addition of CaCl after the first unit of blood components in transfusion protocols.

**References**

1. Ditzel R, Anderson J, Eisenhart W, Rankin C, DeFeo D, Oak S, et al. A review of transfusion- And trauma-induced hypocalcemia: Is it time to change the lethal triad to the lethal diamond? J Trauma Acute Care Surg. 2020; 88(3):434–9.
2. Moore E, Moore H, Kornblith L, Neal M, Hoffman M, Mutch N, et al. Trauma-induced coagulopathy. Nat Rev Dis Prim. 2021;7(1).

## O8 “Pump, pleura, pouring blood?”: The development and introduction of a novel point-of-care ultrasound protocol in a pre-hospital advanced trauma service

### Jamin M Mulvey^1,*^, Shadman Aziz^2^, Salman Naeem^2^, Matthew Mak^1^, Daniel Nevin^1^

#### ^1^London’s Air Ambulance Charity, London, UK. ^2^Barts Health NHS Trust, London, UK

##### *Correspondence: jmulvey@ausdoctors.net

***Scandinavian Journal of Trauma, Resuscitation and Emergency Medicine*** 2023, **31(1)**: O8

**Background: **Point-of-care ultrasound (PoCUS) is increasingly used in the pre-hospital environment for diagnostic and therapeutic purposes in the management of shock [1]. Clinical examination can identify injuries and the aetiology of shock in most patients, however, there may be diagnostic uncertainty, or coexisting pathologies. Lengthy PoCUS examinations are not beneficial in pre-hospital care: they can introduce on-scene delays, loss of situational awareness and clinicians risk task-fixation.

**Methods: **London’s Air Ambulance (LAA) is a physician-paramedic pre-hospital critical care service providing a 24/7 advanced trauma care team in the London urban area, covering approximately 6,731km^2^ and a population of around 12 million. In order to build a PoCUS program at LAA, we required a bespoke, truncated, and rapid protocol, which answers specific questions relevant for pre-hospital trauma care.

**Results: **The “Pump, Pleura and Pouring Blood?” protocol combines aspects of the Extended Focused Assessment using Sonography in Trauma and the Rapid Ultrasound for Shock and Hypotension examinations, but with limited windows to answer questions relevant to our cohort of critically injured patients. Our protocol includes a single subxiphoid view of the heart to look for cardiac tamponade and function (pump); bilateral scans of the anterior chest to look for pneumothorax (pleura); and bilateral scans of the lung bases, with an extended examination of the right upper quadrant to look for haemothorax and haemoperitoneum respectively (pouring blood). LAA clinicians can perform the full or limited protocol to quickly gather information to guide and prioritise resuscitative efforts; including decisions around performing thoracostomy, thoracotomy, volume replacement with blood products, REBOA, and cessation of resuscitation.

**Discussion: **The “Pleura, Pump, Pouring Blood?” protocol is a nuanced, truncated and rapid PoCUS examination developed for use at LAA. Future research is planned to investigate the role and impact of PoCUS on decision-making and patient outcomes at LAA.

**Acknowledgements**

Special thanks go to the London’s Air Ambulance PoCUS Working Group for the protocol development, training, and governance involved with this project.

**Reference**

1. Bøtker MT, Jacobsen L, Rudolph SS, Knudsen L. The role of point of care ultrasound in prehospital critical care: a systematic review. Scandinavian journal of trauma, resuscitation and emergency medicine. 2018; 26:1–4.

## O9 A cross-sectional need and feasibility assessment of prehospital endovascular resuscitation for trauma in New South Wales, Australia

### Andrew Milne^1,*^, Chris Harrington^2^, Jonathon Morris^2^, Alexandra Nicoll^2^, Helen Oliver^2^, Abhinav Singh^2^, Oscar Wigginton^2^, Tom Evans^2^

#### ^1^Trauma Anaesthesia Group, Royal London Hospital, Barts Health NHS Trust, London UK. ^2^Greater Sydney Area Helicopter Emergency Medical Service, New South Wales Ambulance Service, Sydney, Australia

##### *Correspondence: andrew.milne1@nhs.net

***Scandinavian Journal of Trauma, Resuscitation and Emergency Medicine*** 2023, **31(1)**: O9

**Introduction: **Resuscitative balloon occlusion of the aorta (REBOA) enables control of non-compressible sub-diaphragmatic haemorrhage (SH) and has seen prehospital use, including in austere environments [1]. We assessed the need for, and feasibility of, REBOA’s implementation at a helicopter emergency medical service (HEMS) serving urban, rural and remote areas of New South Wales.

**Materials and methods: **The Greater Sydney Area (GSA) HEMS database entries for patients who received a blood transfusion or triggered a major haemorrhage pre-alert between 2018 and 2021 were interrogated. Patients with traumatic SH were assessed by clinicians experienced in REBOA and need for endovascular resusciation (ER) identified based on signs of exsanguination (4 Fr early femoral access [EFA], zone 1 [Z1] or zone 3 [Z3] REBOA). Mission logistics and outcomes were characterised among patients identified as potentially benefitting from REBOA.

**Results: **766 patients were included. 579 (75.6%) had sustained trauma of which 274 (47.3%) had SH. The estimated incidence of ER-amenable injuries was 0.87 (95% CI 0.77 to 0.99) per 100,000 adult population/year. Among these EFA was recommended in 170 (62.0%), Z3 in 19 (6.9%) and Z1 in 45 (16.4%). Z1/Z3 REBOA candidates were predominantly taken to major trauma centres within Sydney (45 [70.3%]) with a median transfer time of 23 (IQR 15–51) minutes. Most incidents occurred in urban areas (31 [48.4%]) with 6 (9.4%) in remote sites. Patients received a median of 3.0 (IQR 3.0–6.5) units of blood products in the prehospital phase and 22.5 units (IQR 16.0–40.5) in hospital. Of the REBOA candidates, 7 (10.9%) died on scene, 9 (14.1%) *en route* or soon after arrival to hospital and 11 (17.2%) on follow up.

**Conclusion: **Potential REBOA candidates were few but suffered high mortality and received large volumes of blood products, paralleling previous work [2]. Mission logistics were not prohibitive, especially with use of partial REBOA [3].

**References**

1. Chan CN, Kadir B, Ahmed Z. The role of prehospital REBOA for hemorrhage control in civilian and military austere settings: a systematic review. *Trauma Care* 2022;2:63–78.
2. Eksteen A, O'Dochartaigh D, Odenbach J, Douma MJ, O'Neill K, Anantha R, Bradley NL, Gauri A, Widder S. A gap analysis of the potential use of resuscitative endovascular balloon occlusion of the aorta (REBOA) in trauma at two major Canadian trauma centers. CJEM 2021;23:36–44.
3. Matsumura Y, Matsumoto J, Kondo H, Idoguchi K, Ishida T, Kon Y, Tomita K, Ishida K, Hirose T, Umakoshi K, Funabiki T; DIRECT-IABO Investigators. Fewer REBOA complications with smaller devices and partial occlusion: evidence from a multicentre registry in Japan. Emerg Med J. 2017;34:793–799.

## O10 Streamlining communication in the stroke care pathway using prehospital National Institutes of Health Stroke Scale (ParaNASPP): a stepped-wedge, cluster-randomised controlled trial

### Helge Bugge^1,*^, Mona Guterud^1,*^, Jo Røislien^1^, Jo Kramer-Johansen^2^, Mathias Toft^3^, Hege Ihle-Hansen^3^, Kristi Bache^4^, Karianne Larsen^1^, Anne-Cathrine Braarud^5^, Else Sandset^3^, Maren R. Hov^3^

*Joint first authors.

#### ^1^Department of Research, Norwegian Air Ambulance Foundation, Oslo, Norway. ^2^Norwegian National Advisory Unit on Prehospital Emergency Medicine (NAKOS) and Air Ambulance Department, Norway. ^3^Department of Neurology, Oslo University Hospital, Norway. ^4^Faculty of Medicine, Institute of Basic Medical Sciences, University of Oslo, Norway. ^5^Division of Prehospital Services, Oslo University Hospital, Norway

##### *Correspondence: helge.bugge@norskluftambulanse.no

***Scandinavian Journal of Trauma, Resuscitation and Emergency Medicine*** 2023, **31(1)**: O10

**Background: **Improved tools for prehospital stroke recognition are recommended [1]. We aimed to improve triage and diagnostic accuracy using prehospital National Institutes of Health Stroke Scale (NIHSS).

**Methods: **ParaNASPP was a stepped-wedge cluster-randomised controlled trial at Oslo University Hospital, Norway. Patients with suspected acute stroke by paramedics were eligible for inclusion. All paramedics started as controls, using standard stroke protocol, before sequential crossover of clusters to intervention every 12th week. The intervention consisted of a mobile application with NIHSS and standardised communication with the in-hospital stroke physician. The primary outcome was the proportion of patients with a final discharge diagnosis of stroke, reported as positive predictive value (PPV). Other outcomes included NIHSS score at admission, time variables, and functional outcome. This trial is registered with ClinicalTrials.gov, NCT04137874.

**Results: **Between 3rd June 2019 and 1st July 2021, 935 patients were included in the trial. 134 were excluded or did not consent to participation, resulting in 447 inclusions in the intervention group and 354 in the control group. There was no difference in PPV, 48·1 (43.4–52.8) in intervention and 45.8 (40.5–51.1) in control (*p* = 0.5). NIHSS score (IQR) was significantly lower in the intervention group (2 (0.0–5.0 vs 2 (0.5–6.5) (*p* = 0.02)). Prehospital on-scene time was five minutes longer in the intervention group (*p* < 0·001), while in-hospital door to computed tomography time was two minutes shorter (*p* = 0.001). Shift in functional outcome was in favour of the intervention with adjusted odds ratio (OR) of 0.65 (0.44–0.95, *p* = 0.03).

**Conclusion: **Implementation of prehospital NIHSS did not improve diagnostic accuracy in patients with suspected stroke. Patients with lower NIHSS scores at admission in the intervention group suggests that milder strokes were identified, also reflected by the better functional outcome in the intervention group.

**Reference**

1. Brandler ES, Sharma M, McCullough F, et al. Prehospital Stroke Identification: Factors Associated with Diagnostic Accuracy. Journal of stroke and cerebrovascular diseases: the official journal of National Stroke Association 2015; 24(9): 2161–6.

## O11 Nasal oropharyngeal suction for evacuation of blood and regurgitation fluids (NOSE-BARF)

### Espen Fevang*, Sandra Larssen Clifford

#### Faculty of Health Sciences, University of Stavanger, Stavanger, Norway

##### *Correspondence: espen.fevang@uis.no

***Scandinavian Journal of Trauma, Resuscitation and Emergency Medicine*** 2023, **31(1)**: O11

**Background: **Blood, vomitus and secretions obstructing and obscuring the airway are amongst the most common challenges in pre-hospital advanced airway management [1]. A simple method successfully used in our system on both drownings and gastrointestinal (GI) bleedings with severe regurgitation into the larynx, is a gastric lavage tube inserted through the nasal cavity and placed in the oropharynx. This specific tube placement seems effective for removing secretions coming from the esophagus, trachea, oral and nasal cavity, while concomitantly facilitating optimal conditions for establishing a definitive airway.

**Case report: **We present a 54-year-old patient with severe upper GI-bleeding due to suspected ruptured esophageal varices. The patient was initially in shock with BP 50/25, pulse 120 and GCS 11. After initial volume resuscitation the patient was prepared for emergency endotracheal intubation (ETI) in the ER, a procedure which was expected to be difficult considering the ongoing massive hemorrhage. A 22-Fr gastric lavage tube was inserted through the nasal cavity, placed in the oropharynx and connected to an activated suction unit. The patient remained in a semiprone position and continuous suction of blood was effective throughout the procedure. ETI was successfully performed on first attempt in less than 15 s, despite heavy bleeding.

**Conclusion: **A GI tube placed in the oropharynx through the nasal cavity may be of advantage when maneuvering challenging airways involving both blood and regurgitation materials. By bypassing the oral cavity, the tube does not interfere with normal airway management, remains in a relatively stable position throughout initial patient treatment, can be connected to a continuous suction device and does not require a dedicated person controlling it during the procedure. Consequently, further studies on the method should be performed to obtain precise indications and limitations.

Written consent to publish has been obtained from the patient’s next-of-kin.

**Reference**

1. Helm M, Hossfeld B, Schafer S, Hoitz J, Lampl L. Factors influencing emergency intubation in the pre-hospital setting-a multicentre study in the German Helicopter Emergency Medical Service. Br J Anaesth. 2006;96(1):67–71.

**Poster presentations**

## P1 Mixed methods contribute to a deeper understanding of interhospital transfer decisions concerning patients with isolated moderate-to-severe traumatic brain injury

### Mathias Cuevas-Østrem^1,*^, Torben Wisborg^2^, Kjetil Thorsen^1^, Olav Røise^3^, Elisabeth Jeppesen^4^

#### ^1^Department of Research and Development, Norwegian Air Ambulance Foundation, Oslo, Norway. ^2^Norwegian National Advisory Unit on Trauma, Division of Emergencies and Critical Care, Oslo University Hospital, Oslo, Norway. ^3^Norwegian Trauma Registry, Division of Orthopaedic Surgery, Oslo University Hospital, Oslo. ^4^Faculty of Health Sciences, University of Stavanger, Stavanger, Norway

##### *Correspondence: mathias.cuevas-ostrem@norskluftambulanse.no

***Scandinavian Journal of Trauma, Resuscitation and Emergency Medicine*** 2023, **31(1)**: P1

**Background: **Isolated moderate-to-severe traumatic brain injury (m-s TBI) is associated with hospitalizations and poor outcomes [1]. Interhospital transfer to neurotrauma centers (NTC) is decisive for patients requiring neurosurgery or neurocritical care primarily admitted to non-specialist acute care trauma hospitals (ACTH). Knowledge of which factors influence transfer decisions is important to improve patient safety and evaluate the trauma system. Trauma registries are valuable sources of information but may fail to capture complexity. Qualitative methods add a deeper understanding of complex processes and are valuable supplements to trauma registry analyses. The aim of this study was to demonstrate how a mixed methods approach was used to examine factors associated with interhospital transfer of patients with isolated m-s TBI.

**Method: **Data from the Norwegian Trauma Registry was collected to identify patients with isolated m-s TBI primarily admitted to ACTH (2015–2020). A multivariable logistic regression model was developed using purposeful selection to identify factors associated with interhospital transfer to NTCs. Four focus group interviews were conducted: two with trauma team leaders (residents) from ACTHs and two with neurosurgeons (residents and consultants) from NTCs, in two regions in Norway. Transcripts were analyzed using thematic analysis [2].

**Results: **The study included 1735 patients with isolated m-s TBI, of which 692 (40%) were transferred to NTCs. Logistic regression model identified age, NISS, GCS, ASA, gender, injury mechanism, and year of injury as factors significantly associated with transfer decisions, while distance between ACTH and NTC and centrality index of injury site was not. Preliminary interview analyses further identified other factors, such as patients’ daily life functions, prognosis, and experience of involved staff.

**Conclusion: **Several factors associated with interhospital transfer of m-s TBI patients were identified by applying a mixed methods approach. Transfer decisions are complex, and focus groups interviews revealed factors not available from trauma registries, highlighting the value of combining methods.

**References**

1. Maas AIR, Menon DK, Manley GT, Abrams M, Akerlund C, Andelic N, Aries M, Bashford T, Bell MJ, Bodien YG, et al. Traumatic brain injury: progress and challenges in prevention, clinical care, and research. Lancet Neurol. 2022.
2. Clarke V, Braun V, Hayfield N. Thematic analysis. Qualitative psychology: A practical guide to research methods. 2015;222(2015):248.

## P2 Pre-oxygenation using high-flow nasal oxygen vs tight facemask in trauma patients undergoing emergency anaesthesia: a prospective, before-and-after, study

### Albin Sjöblom^1,*^, Magnus Hedberg^1^, Malin Jonsson Fagerlund^1^

#### ^1^Perioperative medicine and Intensive care, Karolinska University Hospital, Stockholm, Sweden

##### *Correspondence: Albin.sjoblom@ki.se

***Scandinavian Journal of Trauma, Resuscitation and Emergency Medicine*** 2023, **31(1)**: P2

**Background: **Patients suffering major traumatic injuries frequently require emergency anaesthesia. Due to often compromised physiology and time-sensitive management, trauma patients may be more prone to desaturate during induction of anaesthesia. Apnoeic oxygenation using high-flow nasal oxygen (HFNO) has been shown to increase time until desaturation [1,2]. This study investigated whether pre-oxygenation using HFNO could decrease the risk of desaturation during induction of anaesthesia in trauma patients.

**Material and methods: **This study was conducted at the Karolinska University Hospital, a level-one trauma centre. Adult patients suffering major traumatic injuries in need of emergency anaesthesia at the trauma room or adjacent operating room were included around the clock. The first eight months of enrollment patients were pre-oxygenated using thigh-fitting facemask. HFNO was then introduced as a method for pre-oxygenation of trauma patients. Primary outcome was difference in number of patients desaturating < 93% between the two methods for pre-oxygenation.

**Results: **Data from 95 patients were analysed. Facemask pre-oxygenation was performed in 69 patients while 26 patients were pre-oxygenated with HFNO. The most common indication for anaesthesia was emergency surgery (56%) followed by impaired consciousness (20%). There were no differences in patient characteristics between the groups. Pre-oxygenation (291 s vs 188 s, *p* < 0.001) and apnoea time (88 s vs 80 s, p = 0.015) were longer in the HFNO group. A total of 11 (11.6%) patients desaturated < 93%, eight (11.6%) vs three (11.5%) in the facemask and HFNO group respectively, *p* = 1. No differences in end-tidal O_2_ concentrations in the first breath after intubation could be seen, 82% vs 80%, in the HFNO group and facemask group respectively.

**Conclusions: **In this prospective, before-and-after, study investigating pre-oxygenation in trauma patients undergoing emergency anaesthesia we could not see any difference in number of patients desaturating when pre-oxygenation was performed with HFNO compared to tight-fitting facemask.

**References**

1. Patel A, Nouraei SA. Transnasal Humidified Rapid-Insufflation Ventilatory Exchange (THRIVE): a physiological method of increasing apnoea time in patients with difficult airways. Anaesthesia. 2015;70(3):323–9.
2. Gustafsson IM, Lodenius Å, Tunelli J, Ullman J, Jonsson Fagerlund M. Apnoeic oxygenation in adults under general anaesthesia using Transnasal Humidified Rapid-Insufflation Ventilatory Exchange (THRIVE): a physiological study. Br J Anaesth. 2017;118(4):610–7.

## P3 Comparison of the effects of etomidate versus ketamine as rapid sequence intubation sedatives in adult trauma patients

### Jinjoo Kim^1^, Kyoungwon Jung^1^, Sora Kim^2^, Jayoung Yoo^2^, Seoyoung Song^2^, Yo Huh^1,*^

#### ^1^Division of Trauma Surgery, Department of Surgery, Ajou University School of Medicine, Suwon, Republic of Korea. ^2^Ajou University hospital, Suwon, Republic of Korea

##### *Correspondence: ermdhuhyo@gmail.com

***Scandinavian Journal of Trauma, Resuscitation and Emergency Medicine*** 2023, **31(1)**: P3

**Background: **Etomidate and ketamine are commonly used as sedatives in Rapid Sequence Intubation (RSI) [1], however, there is no consensus on which agent should be favoured in trauma population [2]. The aim of this study is to compare the effects of ketamine versus etomidate on the first pass success and mortality in trauma patients after RSI facilitated emergency intubation.

**Method: **We enrolled and retrospectively reviewed 944 patients who underwent endotracheal intubation in trauma bay at level 1 trauma centre between January 2019 and December 2021. The outcomes were compared between ketamine group and etomidate group after applying propensity score matching in order to balance the overall distribution between two groups.

**Results: **In total 620 patients were included in analysis. Of these patients, 118 patients (19.9%) were administrated ketamine and remaining 502 patients (80.1%) were treated by etomidate. Patients in ketamine group showed significantly higher initial heart rate (105.0 ± 25.7 vs. 97.7 ± 23.6, *p* = *0.003*), more hypotensive (114.2 ± 32.8 mmHg vs. 139.3 ± 34.4 mmHg, *P* = *0.003*), higher GCS (9.1 ± 4.0 vs. 8.2 ± 4.0, *P* = *0.031*), and higher Injury Severity Score (32.5 ± 16.3 vs. 27.0 ± 13.3, *p* < *0.001*) than etomidate group. There was no significant differences in the first pass success rate(90.7% in ketamine group vs. 90.1% in etomidate group, *p* = *0.348)*, mortality (16.1% in ketamine group vs. 20.6% in etomidate group, *p* = *0.348)*, ICU LOS(14.8 ± 31.86 in ketamine group vs. 14.8 ± 15.5 in etomidate group, *p* = *0.998*), ventilator days(9.0 ± 17.9 in ketamine group vs. 9.6 ± 12.1 in etomidate group, *p* = *0.735*) and hospital stay(31.0 ± 38.6 in ketamine group vs. 27.3 ± 22.9 in etomidate group, *p* = *0.322*) in 1:3 propensity score matching analysis.

**Conclusion: **During the trauma resuscitation, ketamine was preferred sedatives for patients with more hemodynamically unstable and consciousness. However, there was no significant difference in clinical outcome.

**References**

1. Matchett G, Gasanova I, Riccio CA, Nasir D, Sunna MC, Bravenec BJ, et al. Etomidate versus ketamine for emergency endotracheal intubation: a randomized clinical trial. Intensive care medicine. 2022;48(1):78–91.
2. Upchurch CP, Grijalva CG, Russ S, Collins SP, Semler MW, Rice TW, et al. Comparison of etomidate and ketamine for induction during rapid sequence intubation of adult trauma patients. Annals of Emergency medicine. 2017;69(1):24–33.

## P4 Excellent agreement of Norwegian Trauma Registry data compared to corresponding data in electronic patient records

### Oddvar Uleberg^1,*^, Natalia Naberezhneva^2^, Marianne Dahlhaug^3^, Vigdis Giil-Jensen^3^, Kjetil G. Ringdal^4^, Olav Røise^3^

#### ^1^Department of Research and Development, Division of Emergencies and Critical Care, Oslo University Hospital, Oslo, Norway. ^2^Biobank and Registry Support Department, Division for medical quality registries for South-Eastern Norway Regional Health Authority, Oslo University Hospital, Oslo, Norway. ^3^National Trauma Registry, Division of Orthopedics, Oslo University Hospital, Oslo, Norway. ^4^Department of Anesthesiology, Vestfold Hospital Trust, Tønsberg, Norway

##### *Correspondence: oddvar.uleberg@stolav.no

***Scandinavian Journal of Trauma, Resuscitation and Emergency Medicine*** 2023, **31(1)**: P4

**Background: **The Norwegian Trauma Registry (NTR) is designed to monitor and improve the quality and outcome of trauma care delivered by Norwegian trauma hospitals. High-quality data recorded in the registry is essential for the trust and use of data. This study aims to perform a data quality check of a subset of core data elements in the registry by assessing agreement between data in the NTR and corresponding data in electronic patient records (EPRs).

**Method: **We validated 49 of the 118 variables registered in the NTR by comparing those with the corresponding ones reviewed in the electronic patient records for 180 randomly selected patients with trauma diagnosis in 2019 at eight public hospitals. Agreement was quantified by calculating observed agreement, Cohen’s kappa and Gwet’s first agreement coefficient (AC_1_) with 95% confidence intervals for 27 nominal variables, quadratic weighted Cohen’s kappa and Gwet’s second agreement coefficient (AC_2_) for five ordinal variables. For nine continuous, one date and seven time variables, we calculated intraclass correlation coefficient (ICC).

**Results: **Almost perfect agreement (AC_1_ /AC_2_/ICC > 0.80) was observed for all examined variables. Nominal and ordinal variables showed Gwet’s agreement coefficients ranging from 0.85 to 1.00. Although there were detected high values of intraclass correlation coefficient (ICC) ranging from 0.88 to 1.00 for continuous and time variables, we found high percentage of missing data for three continuous variables *(Pre-hospital SBP, Pre-hospital RR, Base excess*) and two time variables (*Time until first CT*, *Time until pelvic x-ray*).

**Conclusions: **All the variables assessed in the Norwegian Trauma Registry displayed excellent agreement with the corresponding ones reviewed in electronic patient records. The results of this study show that, with the use of certified registrars and systematic quality assurance of registration, this registry has accurate and valid data for research and quality improvement work.

## P5 Lessons from 6 years of prehospital burn care from Emergency Medical Retrieval and Transfer Service (EMRTS) Wales

### Alistair Gales^1,*^, Owen McIntyre^1,2^, David Rawlinson^2^, Stuart Gill^2^

#### ^1^Welsh Centre for Burn Care, Morriston, Swansea Bay University Health Board, Wales. ^2^Emergency Medical Retrieval and Transfer Service, Wales

##### *Correspondence: Alistair.gales@nhs.net

***Scandinavian Journal of Trauma, Resuscitation and Emergency Medicine*** 2023, **31(1)**: P5

**Background: **Major burn injuries make up over 5% of all traumatic injuries across the United Kingdom. 1 Appropriate and timely pre-hospital care can significantly affect mortality and morbidity from burn injuries. The Emergency Medical Retrieval and Transfer service (EMRTS) in Wales attend around 35 primary burns missions annually.

**Method: **This retrospective national service evaluation included all Burns patients treated by EMRTS over 6 years since its foundation in 2015. Care was audited against current best practice guidelines from the British Burns Association and Faculty for Pre-Hospital Care 2 including management of airway, smoke inhalation injury, burn size estimation, fluid resuscitation, temperature and analgesia.

**Results: **A total of 168 primary burns missions were reviewed. There were 25 intubated burns patients with a 95% first time intubation success rate, compared with 89.9% in the general EMRTS population. There were no failed intubations from a mixed group of critical care practitioners, PHEM trainees and consultants. 30 patients had evidence of smoke inhalation with 7 patients meeting criteria for prehospital administration of Hydroxocobalamin with intermediate or severe cyanide toxicity. 3 In this cohort smaller burns tended to be overestimated by 2.5% (*p* = 0.0009) compared to burns over 15% total body surface area which were underestimated by 5.3% (*p* = 0.014). Fluid resuscitation recommendations were met in only 50% (*p* = <0.0001) of patients. Analgesia was provided with mainly opiates and ketamine. The use of targeted temperature, burns first aid and cooling measures were inconsistently reported.

**Discussion: **In this series of pre-hospital burns patients airway management using existing guidelines resulted in a high first time success rate for intubation in severe burn injuries. A cohort was identified in whom pre-hospital Hydroxocobalamin was indicated which has led to a change in practice within EMRTS. Prehospital burn estimation and appropriate prehospital fluid resuscitation remain extremely difficult to achieve and research is required to improve this further.

**References**

1. Kalson NS, Jenks T, Woodford M, et al. (2012) Burns represent a significant proportion of the total serious trauma workload in England and Wales. Burns. May 31;38(3):330–9.
2. E Battaloglu, L Greasley, J Leon-Villapalos, A Young, K Porter. Management of Burns in Pre—Hospital Trauma Care. Faculty of Pre—Hospital Care &amp; British Burn Association. Expert Consensus Meeting. 2019 [cited 2022 Oct 14]. Available from: https://fphc.rcsed.ac.uk/media/2621/burns-consensus-2019.pdf
3. Anseeuw K, Delvau N, Burillo-Putze G, De Iaco F, Geldner G, Holmström P, Lambert Y, Sabbe M. Cyanide poisoning by fire smoke inhalation: a European expert consensus. European Journal of Emergency Medicine. 2013 Feb 1;20(1):2–9.

## P6 Piloting a multi-discipliary follow up clinic for patients discharged from a Major Trauma Centre with isolated blunt thoracic injuries: A Quality Improvement project

### Natalie Hildebrand*, Alexandra Buckland, Jennifer Illingworth, George Peck, Davina Richardson

#### Major Trauma Department, St. Mary’s Hospital, Imperial College Healthcare NHS Trust, London, UK

##### *Correspondence: nataliehildebrand@nhs.net

***Scandinavian Journal of Trauma, Resuscitation and Emergency Medicine*** 2023, **31(1)**: P6

**Background: **Blunt thoracic injury accounts for more than 15% of UK trauma admissions and is associated with substantial sequelae that can impact on daily functioning [1]. Extensive work has been completed on inpatient rib fracture pathways across the Major Trauma system. However patients are not routinely reviewed following discharge. We piloted whether a follow up clinic at 6–8 weeks following a patients’ discharge would identify any unmet needs.

**Methods: **During a 10 month period, patients with isolated blunt thoracic injury admitted to the Major Trauma Centre (MTC) at St Mary’s Hospital, were identified and consented for follow up. The Trauma therapists and Trauma Coordinators completed screening telephone calls at 6–8 weeks following a patients’ discharge. An EQ5D-3L, patient reported health scores and patient analgesia regime were documented. Patients with ongoing health or therapy needs were invited to a clinic with a relevant medical professional. Any further unmet needs were addressed through referrals to appropriate healthcare professionals.

**Results: **127 eligible patients were identified between May 2021 and March 2022. 69 of these patients were contactable for a screening call of which 13 required input from a medical professional at clinic. The main symptom reported from screening calls and attendance at clinic was ongoing pain. Further management of this could usually be addressed over the phone with reassurance, advice regarding analgesia management and general paced activity advice. 4 patients had ongoing unmet musculoskeletal needs for which they received referrals to their GP, physiotherapists or fracture clinic.

**Conclusion: **Further information for patients regarding pain patterns at the point of discharge may provide some reassurance. Advice regarding weaning of opioid pain medication in discharge summaries may be beneficial. Further research is required to specifically identify whether previously employed patients have been able to return to work following their blunt thoracic trauma.

**Reference**

1. Baker E, Xyrichis A, Norton C, Hopkins P, Lee G. The long-term outcomes and health-related quality of life of patients following blunt thoracic injury: a narrative literature review. Scandinavian Journal of Trauma, Resuscitation and Emergency Medicine. 2018 26:67

## P7 Pre-hospital Early Femoral Access (EFA) at London’s Air Ambulance: a service evaluation

### RM Greenhalgh^1,2,*^, S Jefferys^3^, L Wynne^2^, K Acheson^4^, N Hudson-Peacock^5^, L Kocierz^1,2^, S Sadek^1,2^, Z.B. Perkins^1,2^, R.A. Lendrum^1,2^

#### ^1^London’s Air Ambulance Charity, London, UK. ^2^Barts Health NHS Trust, London UK. ^3^St Mary’s Hospital, Imperial College Healthcare NHS Trust. ^4^St George’s University Hospitals NHS Foundation Trust. ^5^King College London Hospital NHS Foundation Trust

##### *Correspondence: Robert.Greenhalgh3@nhs.net

***Scandinavian Journal of Trauma, Resuscitation and Emergency Medicine*** 2023, **31(1)**: P7

**Background: **Early Femoral Access (EFA) is a potentially valuable monitoring adjunct for critically unwell trauma patients at London’s Air Ambulance. EFA enables intra-arterial blood pressure (IABP) monitoring and as a bridge to endovascular resuscitation techniques, including resuscitative endovascular balloon occlusion of the aorta (REBOA).

**Methods: **A prospective audit of patients who underwent EFA by London’s Air Ambulance between August 2019 and November 2021. EFA is performed by inserting a 4Fr catheter under ultrasound guidance (US) into the common femoral artery (CFA). Procedural complications and outcome data were collected.

**Results: **During the 2-year period, 97 EFA arterial lines were attempted. Median patient age was 35 (IQR 26–50). The mechanism of injury of patients who had EFA attempts included penetrating trauma (29%), road traffic collision (24%), fall from height (23%), patient under train (12%), medical (7%), burns or electrocution (3%) and head injury (2%). The commonest indication included haemodynamic monitoring in potential REBOA patients 50/97 (52%). EFA was successful in 82/97 (85%) attempts with a first attempt success rate of 77/97 (79%). Using radiological evidence, the arterial puncture point was identified retrospectively in 67 patients: 53 (79%) in the common femoral artery, 11 (16%) in the external iliac artery and 3 (4%) in the superficial femoral artery. The commonest reason for failure was the inability to visualise the CFA on US (8/15; 53%). Associated complications were followed up in 84 patients with 6 complications identified. Specifically, 2/84 (2.4%) localised intimal flap dissection, 1/84 (1.2%) pseudoaneurysm, 1/84 (1.2%) retroperitoneal haemorrhage, 1/84 (1.2%) peritoneal breach and 1/84 (1.2%) line sepsis.

**Conclusions: **Pre-hospital EFA can be performed with a high success rate and a low complication rate, similar to that seen in an in-hospital setting. Meticulous US guided arterial cannulation technique in an optimised pre-hospital environment is fundamental to minimise the incidence of non-CFA placement and vascular trauma.

## P8 Standardised mortality rates for non-traumatic deaths among survivors of trauma in Norway (2015–2018)

### Anders Rasmussen^1,*^, Jo Stenehjem^2^, Olav Røise^3^, Jon Michael Gran^4^, Thomas Kristiansen^5^, Oddvar Uleberg^5^, Leiv-Arne Rosseland^5^, Trond Nordseth^5^

#### ^1^Department of Anaesthesia, Innlandet Hospital Trust, Hamar, Norway. ^2^Department of Research, Cancer Registry of Norway, Oslo, Norway. ^3^Institute of Clinical Medicine, University of Oslo, Oslo, Norway. ^4^Institute of Basic Medical Sciences, University of Oslo, Oslo, Norway. ^5^Department of Research and Development, Division of Emergencies and Critical Care, Oslo University Hospital, Oslo, Norway

##### *Correspondence: Anders.Rasmussen@sykehuset-innlandet.no

***Scandinavian Journal of Trauma, Resuscitation and Emergency Medicine*** 2023, **31(1)**: P8

**Background: **Morbidity and mortality after trauma constitute major burdens for individuals and society. Adversity in life, health related problems and inequality are possible risk factors for a possible excess in non-trauma related mortality following traumatic injury. The aim of this study was to assess standardized mortality rates (SMR) for non-trauma related death.

**Methods: **To characterize the challenges met by survivors of traumatic injury in Norway, the Injury Prevention and Outcomes Following Trauma (IPOT) study combine data from the National Trauma Registry (NTR) with five other national registries. Patients in NTR were included between 1st of January 2015 and 31st of December 2018. Data from the National Cause of Death registry was applied to assess registered cause of death for the patients included, and for all deaths in Norway in the period studied (the reference population). Standardized mortality ratios (SMRs) with 95% confidence intervals (CIs) were computed by using sex, age, and period-specific reference rates from the Cause of Death registry. Trauma related deaths (ICD-10 chapters S, T, V, W, X and Y) were excluded both in the trauma population and in the reference population.

**Results: **A total of 26,562 patients were included in the study, of whom 962 patients were registered with a trauma-related cause of death. There were 481 deaths among 17,265 men, and 388 deaths among 8335 women in whom a non-traumatic cause of death was registered. The expected number of non-trauma related deaths were 462 and 336, respectively. The estimated SMR for men was 1.04 (95% CI: 0.95–1.14), and for women 1.15 (95% CI: 1.04–1.15) for the period studied.

**Conclusion: **We found excess death among female trauma patients for non-trauma related causes of death.

## P9 Cooperation creating capacity—integration of HEMS and critical care transfer teams within the Welsh trauma system

### Thomas Hirst^1,*^, Michael Slattery^1^, Meryl Jenkins^1^, Ben Seabourne^2^, David Rawlinson^2^

#### ^1^Adult Critical Care Transfer Service Cymru, Cardiff, UK. ^2^Emergency Medical Retrieval and Transfer Service, Dafen, UK

##### *Correspondence: tom.hirst@wales.nhs.uk

***Scandinavian Journal of Trauma, Resuscitation and Emergency Medicine*** 2023, **31(1)**: P9

**Background: **In Wales, large distances between hospitals and remote population centres uniquely complicate delivery of networked trauma care. Several hour journeys to definitive treatment from scene of incident mean secondary transfer from local hospitals is often required. We describe cooperation between our adult critical care transfer service (ACCTS) and our advanced prehospital team, the Emergency Medical Retrieval and Transfer Service (EMRTS) in overcoming the challenges to delivering timely care to critically injured patients in Wales.

**Methods: **We conducted a review of electronic records for trauma patients transferred between hospitals by the ACCTS and EMRTS teams from the launch of ACCTS on August 16 2021 to October 26 2022.

**Results: **Over 436 days there were 131 adult secondary trauma transfers. 69% were conducted by ACCTS and 31% were conducted by EMRTS. Specialist practitioners from EMRTS supported ACCTS in 5 joint transfer missions. 80 patients were transported to a major trauma centre (MTC), 46 patients were repatriated to a local unit and 5 patients required transfer for specialist non-MTC care. 46 patients were deemed to require hyperacute surgery, 27 were deemed to require emergency transfer and 7 were deemed to require less urgent specialist MTC care. During 55% urgent ACCTS missions the EMRTS team were activated to respond to another incident, and 7% of ACCTS missions occurred at night when one EMRTS team covers all of Wales.

**Conclusions: **Timely movement both in and out of the major trauma centre is essential to allow effective treatment to the population within a major trauma network. Our data show how a critical care transfer team can allow effective escalation and earlier repatriation of major trauma patients, creating network capacity, while supporting our primary HEMS service to maximise their availability for taskings. Further work is needed to characterise the patients transferred by each service to aid triage and inform future service development.

## P10 Patient reported outcomes from the Norwegian Trauma Registry

### Nina G. Andersen^1,*^, Marianne Dahlhaug^2^, Olav Røise^2^, Trond Nordseth^3^, Leiv Arne Rosseland^3^, Oddvar Uleberg^3^

#### ^1^Department of Anaesthesiology, Division of Emergencies and Critical Care, Oslo University Hospital, Oslo, Norway. ^2^The Norwegian Trauma Registry, Division of Orthopaedic Surgery, Oslo University Hospital, Oslo, Norway. ^3^Department of Research and Development, Division of Emergencies and Critical Care, Oslo University Hospital, Norway

##### *Correspondence: nina.gjerde.andersen@gmail.com

***Scandinavian Journal of Trauma, Resuscitation and Emergency Medicine*** 2023, **31(1)**: P10

**Background: **Return to work or education after trauma is recommended as a measure of long-term functional level [1], indicating the ability to combine both physical and mental skills in performing complex and compound tasks. Patient Reported Outcome Measures (PROMs) can be used to assess a patient’s health related quality of life after trauma. Since July 2021 patients in the Norwegian Trauma Registry (NTR), injured after 01.01.21 have been invited to answer the EQ-5D-5L questionnaire.

**Method: **Trauma survivors included in the NTR throughout 2021 were invited to answer the EQ-5D-5L questionnaire and supplementary questions about previous illness, work ability pre- and post-injury, and received treatment six months after trauma. The EQ-5D-5L comprises five dimensions: mobility, self-care, usual activities, pain/discomfort, and anxiety/depression. Each dimension has five levels from no problems to extreme problems. We extracted data from patients, who at the time of trauma, were employed or in education, and investigated their health-related quality of life and change in work ability or education six months after trauma.

**Results: **A total of 2284 questionaries were sent out. Prior to trauma, 587 patients were employed or in education. After six months, 484 (82%) patients had returned to work or education. Severe pain or discomfort was reported in 24% of those not returning to work/education compared to 2.5% of those returning. The majority (62%) of those returning to work/education had no problems doing usual activities (e.g., housework, leisure) compared to those not returning (16%).

**Conclusion: **By implementing PROMs into NTR we gain a detailed understanding of trauma survivors’ quality of life, their return to work or education, and long-term outcome in general; information that may improve quality of future individual patient follow-up.

**Acknowledgement**

The authors thank the national registrars for their contributions.

**Reference**

1. Ardolino A, Sleat G, Willett K. Outcome measurements in major trauma–results of a consensus meeting. Injury. 2012;43(10):1662–6.

## P11 Haemodynamic effects of supplemental oxygen during simulated haemorrhage in healthy volunteers: a randomised, controlled, double-blind crossover study

### Sole Lindvåg Lie^1,*^, Jonny Hisdal^2^, Marius Rehn^1^, Lars Øivind Høiseth^3^

#### ^1^Department of Research and Development, Norwegian Air Ambulance Foundation, Oslo, Norway. ^2^Faculty of Medicine, University of Oslo, Oslo, Norway. ^3^Department of Anaesthesiology and Intensive Care, Division of Emergencies and Critical Care, Oslo University Hospital, Oslo, Norway

##### *Correspondence: sole.lindvaag.lie@norskluftambulanse.no

***Scandinavian Journal of Trauma, Resuscitation and Emergency Medicine*** 2023, **31(1)**: P11

**Background: **Trauma patients often receive high inspired fractions of supplemental oxygen despite the evidence being scarce (1), and there is a need for more knowledge about the effect of oxygen on systemic haemodynamics during haemorrhage. We therefore aimed to study the effects of supplemental oxygen on the systemic haemodynamic response to simulated haemorrhage, with change in cardiac output (CO) as the primary outcome. Study registered in ClinicalTrials.gov: NCT05150418. The protocol was published in advance (2).

**Method: **Fifteen healthy volunteers received 100% oxygen or air on two separate days during simulated haemorrhage by lower body negative pressure (LBNP) (3) in a double-blinded crossover design. To simulate progressive haemorrhage LBNP was increased every 3 min in steps of 10 mmHg from 0 to 80 mmHg or until haemodynamic decompensation. Oxygen and air were administered via a reservoir face mask at 15 L/min. Non-invasive mean arterial blood pressure (MAP) was measured by Nexfin, heart rate (HR) by ECG, stroke volume (SV) by suprasternal Doppler ultrasound and CO calculated as the product of HR and SV. The effect of oxygen compared to air on changes in the haemodynamic variables was examined using mixed regression.

**Results: **Supplemental oxygen had no statistically significant effect on the changes in CO per LBNP-level (0.031 L/min, 95% confidence interval [CI]: -0.015 to 0.077, *P* = 0.188), SV (0.39 mL, 95% CI: -0.39 to 1.2, *P* = 0.331) or MAP (0.072 mmHg, 95% CI: -0.30 to 0.45, *P* = 0.707). A small and statistically significant effect on HR (-0.90 bpm, 95% CI: −1.7 to −0.12, *P* = 0.025) was found, corresponding to a 7 bpm decrease at LBNP 80.

**Conclusion: **Supplemental oxygen had a minor effect on HR, but no other effects on the systemic haemodynamic response to simulated haemorrhage in healthy volunteers. Our findings neither support, nor refute the use of liberal oxygen therapy in haemorrhage.

**References**

1. Eskesen TG, Baekgaard JS, Steinmetz J, Rasmussen LS. Initial use of supplementary oxygen for trauma patients: a systematic review. BMJ Open. 2018 Jul 1;8(7):e020880.
2. Lindvåg Lie S, Hisdal J, Rehn M, Høiseth LØ. Effects of supplemental oxygen on systemic and cerebral hemodynamics in experimental hypovolemia: Protocol for a randomized, double blinded crossover study. PLoS One. 2022;17(6):e0270598.
3. Goswami N, Blaber AP, Hinghofer-Szalkay H, Convertino VA. Lower Body Negative Pressure: Physiological Effects, Applications, and Implementation. Physiological reviews. 2018/12/13 ed. 2019 Jan 1;99(1):807–51.

## P12 How far do you go? Dispatch of ground-based pre-hospital critical care resources

### George Hamson^1^, Rebecca Miller^1,*^, Matthew O’Meara^2^

#### ^1^University of Nottingham Medical School, UK. ^2^Essex & Herts Air Ambulance, UK

##### *Correspondence: mzyrsm@outlook.com

***Scandinavian Journal of Trauma, Resuscitation and Emergency Medicine*** 2023, **31(1)**: P12

**Background: **The need for pre-hospital critical care is present 24/7. Given the high cost and risk associated with aircraft use, many enhanced care teams use ground-based rapid response vehicles during night hours. However, the use of emergency blue light vehicles still carries considerable risk to both the general public and health care professionals [1,2]. We investigated the dispatch of ground-based critical care resources to incidents with increasing transit intervals, to assess the risk-benefits of prolonged blue light driving.

**Method: **We conducted a retrospective study looking at the dispatch of Essex and Herts Air Ambulance (EHAAT) critical care cars over a 5 year period, using pre-existing data on HEMSbase. Successful outcome was defined as a HEMS team conveying a patient, providing treatment or assisting on scene.

**Results: **2727 incidents were included. 81 jobs were > 45 min, 631 jobs were 30–45 min and 2015 jobs were < 30 min transit. Jobs within the 30–45 min category had the highest rate of successful outcome at 94.9% with < 30 min (92.5%) and > 45 min (90.1%) respectively. There was a significant difference (*p* = 0.038) between the rates of stand down between the varying transit times. There was no significant difference (*p* = 0.26) between the extremes of transit interval (> 45 min or < 45 min) but there was a significant difference (*p* = 0.02) between extremes of transit time and jobs 30–45 min away.

**Discussion: **This study has shown a significant difference between the rate of stand down and thus rate of interventions between varying transit times. Teams are stood down in greater proportion at the extremes of travel times. This could represent over-dispatch for nearby incidents and land crews electing to leave scene for prolonged incidents. Geospatial analysis identified hotspots for missions where transit time was > 45. The possibility of stationing additional assets in such hotspots would reduce the risks associated with prolonged blue light drives.

**References**

1. Bentley, M. A. and Levine, R. (2016) 'A National Assessment of the Health and Safety of Emergency Medical Services Professionals', Prehosp Disaster Med, 31(S1), pp. S96-S104.
2. Slattery, D. E. and Silver, A. (2009) 'The hazards of providing care in emergency vehicles: an opportunity for reform', Prehosp Emerg Care, 13(3), pp. 388–97.
3. Rehn, M., Davies, G., Smith, P. and Lockey, D., (2017) ‘Emergency versus standard response: time efficacy of London’s Air Ambulance rapid response vehicle’, EmergMedJ, 34(12), pp806-809

## P13 The unpredictable recovery pathway: A qualitative longitudinal study among physical trauma survivors 18 months after injury

### Jeanette Finstad^1,*^, Thomas Clausen^2^, Olav Røise^3^, Leiv Arne Rosseland^1^, Ingrid Amalia Havnes^4^

#### ^1^Department of Research and Development, Oslo University Hospital, Oslo, Norway. ^2^Norwegian Centre for Addiction Research, Institute of Clinical Medicine, University of Oslo, Norway. ^3^Norwegian Trauma Registry, Division of Orthopedic Surgery, Oslo University Hospital, Oslo, Norway. ^4^Division of Mental Health and Addiction, Oslo University Hospital, Oslo, Norway

##### *Correspondence: uxjefi@ous-hf.no

***Scandinavian Journal of Trauma, Resuscitation and Emergency Medicine*** 2023, **31(1)**: P13

**Background: **Physical trauma continues to significantly contribute to global disability, and many trauma survivors sustain mental and physical impairments, including long-term pain, several years after the injury. There is a knowledge gap on factors that may influence long-term health outcomes. Therefore, this study aimed to explore trauma patients' experiences of the long-term recovery pathway during 18 months post-discharge.

**Methods: **In this qualitative longitudinal study, 13 trauma patients with injuries associated with pain were interviewed six weeks and 18 months after discharge from the trauma centre. The semi-structured interviews were recorded, transcribed verbatim and analyzed thematically.

**Results: **Compared to the subacute phase six weeks post-discharge, several participants reported exacerbated mental and physical health, including increased pain during 18 months following discharge. This, and alternating periods of deteriorated health status during recovery, made the pathway unpredictable. At 18 months post-discharge, participants were coping with experiences of reduced mental and physical health and socioeconomic losses. Four related main themes were identified: (1) the need for follow-up without receiving it, (2) adjusting to a new life situation, (3) suffering mental distress without getting access to mental health care, and (4) coping with persistent pain and reduced physical function. Moreover, at 18 months post-discharge five participants used opioids to relieve chronic somatic pain, relieve mental distress, and reduce withdrawal symptoms. Alcohol was also used to cope with a difficult life situation during recovery.

**Conclusions: **The patients' experiences in this study suggest that subacute health status at discharge from the trauma centre is a poor predictor of long-term outcomes. The participants struggled with biopsychosocial health problems during recovery without being met. Therefore, access to multidisciplinary follow-up outpatient clinics and closer collaboration with primary health services may provide long-term individualized health care, and future studies should explore whether this will improve long-term health outcomes for trauma patients.

## P14 Blunt aortic injury over the years: a single centre experience

### Shehr-Bano Syed^1,*^, Callum Twohig^2^, Ross Davenport^3^, Susan Cross^4^

#### ^1,*^Barts & The London School of Medicine and Dentistry, London, UK. ^2^ACCS Anaesthesia ST1, Croydon University Hospital NHS Trust, London, UK. ^3^Vascular surgeon, Royal London Major Trauma Centre, Bart’s Health NHS Trust, UK. ^4^Consultant Radiologist, Royal London Major Trauma Centre, Bart’s Health NHS Trust, UK

##### *Correspondence: ha18108@qmul.ac.uk

***Scandinavian Journal of Trauma, Resuscitation and Emergency Medicine*** 2023, **31(1)**: P14

**Background: **Immediate outcomes for patients with blunt aortic injury (BAI) have improved due to changes in imaging and treatment guidelines however patient outcomes over the long term have not been studied and there is no consensus regarding surveillance duration and frequency. The aim of this study was to capture how BAI is managed and surveilled at our institute, through assessment of patient outcomes and long term follow up.

**Methods: **Data was collected retrospectively from the trauma registry for patients presenting with BAI from 2012 to 2021. The outcomes of interest examined were: (1) survival to discharge, (2) spinal cord ischaemia, (3) operative intervention, (4) time of intervention from injury, (5) associated injuries, (6) follow-up attendance for imaging and clinic, and (7) changes detected on CTA.

**Results: **A total of 36 patients were included in this study. The majority of patients survived to discharge (n = 32). One patient (n = 1) had evidence of spinal cord ischaemia on imaging within a month of intervention. Sixty four percent of patients (n = 23) required an intervention of which 87% were endovascular with only three open procedures. Most interventions (n = 19) were performed within 24 h. The most common associated injuries were pneumothoraces (n = 23), intra-abdominal injuries (n = 21) and head injuries (n = 19). A repeat scan was only recorded in 83% with wide discrepancies in how frequently scans were repeated, and poor attendance at surveillance clinic with 52% never being seen. Changes detected on repeat imaging included coeliac dissection (n = 1), vertebral steal (n = 1), aneurysms (n = 1), and endoleak (n = 1).

**Conclusion: **This study highlights a significant number of patients are not receiving surveillance imaging or clinic follow up. The reasons for this were not captured but process improvements are required to ensure a robust surveillance strategy is offered to all patients to detect abnormal findings which may require intervention.

## P15 The epidemiology of major trauma patients in Nova Scotia with burn injuries

### James Nunn^1,*^, Jack Rasmussen^2^, Mete Erdogan^3^, Nelofar Kuresh^4^, Robert Green^3^

#### ^1,*^Emergency Medicine, Dalhousie University, Halifax, Canada. ^2^Plastic Surgery, Dalhousie University, Halifax, Canada. ^3^Trauma Nova Scotia, Halifax, Canada. ^4^Neurosurgery, Dalhousie University, Halifax, Canada

##### *Correspondence: james.nunn@dal.ca

***Scandinavian Journal of Trauma, Resuscitation and Emergency Medicine*** 2023, **31(1)**: P15

**Background: **Our objectives were to describe the epidemiology, trends, and predictors of mortality in major trauma patients with burn injuries in Nova Scotia.

**Methods: **Retrospective observational cohort study of all major trauma patients between April 1, 2001, and March 31, 2019, with burn injuries. Data was collected from the Nova Scotia Trauma Registry. Characteristics and outcomes for various patient subgroups were compared using t-tests, chi-square analysis and Fisher’s exact tests. Trend analysis for mortality and other outcomes was performed using linear regression. A multivariate regression model was created to assess for characteristics associated with non-survival.

**Results: **Overall, 436 patients were included in the analysis. Patients predominantly had isolated burns (87.2%, 380/436), major burns (69.7%, 304/436), and associated inhalation injury (60.3%, 263/436). Mean total body surface area (TBSA) of burn was 53.2 ± 35.4%. Overall, 65.6% (286/436) of patients died. We observed a decreasing trend in the overall crude mortality rate (*p* = 0.042) over the study period. Survivors tended to be younger (35.9 ± 20.6 years versus 50.8 ± 23.3 years, *p* < 0.001), male (79.3% versus 65.4%, *p* = 0.002), had lower mean % TBSA involved (29.5 ± 19.9 vs. 67.9 ± 34.9, *p* < 0.001) and had combined trauma (18.7% versus 9.8%, *p* = 0.008). Non-survivors were more likely to have inhalation injuries (75.2% versus 32.0%, *p* < 0.001). Mortality was associated with major burns (OR 8.86, 95% CI 2.45–32.07), inhalation burns (OR 3.42, 95% CI, 1.46–7.97), initial carboxyhemoglobin (OR 1.09, 95% CI 1.05–1.13), and increasing age (OR 1.04, 95% CI 1.02–1.07). Intubation at the scene or in the emergency department was associated with survival (OR 0.084, 95% CI 0.033–0.21).

**Conclusion: **In this population-based study, burn trauma mortality was associated with older age, major burns, inhalational burns, and initial carboxyhemoglobin level. These results provide a foundation of evidence to guide future research and injury prevention strategies to reduce morbidity and mortality from burn injuries in our population.

## P16 E-scooter related injuries: a two-year analysis in Portuguese emergency medical system

### Pedro Vasconcelos^1,*^, João Lourenço^1^, Filipa Barros^1^

#### ^1,*^Instituto Nacional de Emergência Médica (INEM), Lisboa, Portugal

##### *Correspondence: pedro.vasconcelos@inem.pt

***Scandinavian Journal of Trauma, Resuscitation and Emergency Medicine*** 2023, **31(1)**: P16

**Background: **Despite electric scooters (e-scooter) being popular in actual urban mobility settings there is still few evidence concerning the trauma risk and associated severity.

**Methods: **Retrospective review of 2020 and 2021 Portugal prehospital trauma clinical that included written reference to e-scooter. Demographics, mechanism of injury and the injury severity measured with the Revised Trauma Score (RTS) were analysed.

**Results: **From 416 pre-hospital clinical trauma records with the word e-scooter, 158 were excluded due to incomplete data. The analysed sample (n = 258) 53% (n = 137) were male, 47% (n = 121) were female. The average age was 27 years old. Incidence was higher in major cities namely Lisboa 59% (n = 151), followed by Porto 10% (n = 26) and Setúbal 5% (n = 14). This time window showed that 49.7% (n = 128) of the accidents occurred between 2 and 10 pm, and 34.8% (n = 90) occurred during the weekend or holydays. Concerning the mechanism of injury falls accounted for 74% (n = 192), followed by collisions with 18% (n = 46). Accidents in which pedestrian were hit by e-scooters were found in 8% (n = 20) of the clinical records. The clinical analysis revealed extremity trauma in 69% (n = 178), traumatic head injury in 36% (n = 93) and face trauma in 31% (n = 81) of the patients. In this sample 97.3% of the cases had a low severity score (RTS < 12), 1.9% presented with RTS = 11 and only 0.8% with an RTS ≤ 10.

**Discussion: **This retrospective analysis reveals a low prevalence of severe trauma related to e-scooter accidents, nevertheless relevant not only as an alert to an emerging cause of trauma but also as a base of work to road safety authorities to adequate measures of prevention, regulation and penalization to mitigate e-scooter accidents.

**References**

1. Reito A, Öljymäki E, Franssila M, Mattila VM. Incidence of Electric Scooter-Associated Injuries in Finland from 2019 to 2021. JAMA Netw Open. 2022;5(4):E227418.
2. Toofany M, Mohsenian S, Shum LK, Chan H, Brubacher JR. Injury patterns and circumstances associated with electric scooter collisions: A scoping review. Inj Prev. 2021;27(5):490–9.
3. Coelho A, Feito P, Corominas L, Sánchez-Soler JF, Pérez-Prieto D, Martínez-Diaz S, et al. Electric scooter-related injuries: A new epidemic in orthopedics. J Clin Med. 2021;10(15):4–11.

## P17 The PreMeFen protocol: a non-inferiority, open-label, randomized controlled trial in the pre-hospital environment comparing three regimens: inhalational methoxyflurane; intranasal fentanyl; and intravenous morphine

### Randi Simensen^1^, Lars Olav Fjose^1^, Liv -Jorunn Smalberget^1^, Jostein Hagemo^2^, Marius Rehn^2^, Fridtjof Heyerdahl^2,*^

#### ^1^Division of Pre-hospital Services, Innlandet Hospital Trust, Moelv, Norway. ^2^Division of Prehospital Services, Oslo University Hospital, Oslo, Norway

##### *Correspondence: fridtjof.heyerdahl@medisin.uio.no

***Scandinavian Journal of Trauma, Resuscitation and Emergency Medicine*** 2023, **31(1)**: P17

**Background: **Intravenous Morphine has been the mainstay in pre-hospital analgesia, but with certain challenges (1). Fear of opioid adverse events; problems establishing IV access; few non-invasive analgesics; non-physicians setting; and lack of experience represent some of the obstacles to proper pain management. Non-invasive analgesics have been introduced in the ambulance service (2), but with little evidence. The aim of this study is therefore to assess whether inhalational methoxyflurane or intranasal fentanyl is as good as intravenous morphine in reducing Pain Numeric Rating Scale (NRS). The study is on-going, and we here present the study protocol and setup.

**Method: **The protocol follows the Norwegian Norcrin templates for randomized controlled trials, registered in EudraCT (2021–000549-42 NO) and clinicaltrials.gov (NCT05137184), and approved by The Norwegian Medicines Agency and the Regional Ethics Committee.

**Results: **The PreMeFen protocol describes a non-inferiority, open-label, three-arm RCT in the Norwegian EMT-staffed ground ambulance service. Adult patients with a NRS pain score of 4 or more are randomized to one of three regimes: inhaled methoxyflurane; intranasal fentanyl; or intravenous morphine, where each drug is titrated to effect. The primary endpoint is NRS pain score reduction at 10 min after start of treatment.

**Discussion: **The design was chosen because a non-inferiority of the non-intravenous drugs would add these to the analgesic “menu” in the pre-hospital setting, with earlier and more practical administration of pain management in the ambulance. By comparing treatment regimens with titration rather than effect of single doses, the study focuses on proper pain management and will answer pragmatically and realistically whether the methods are good enough for this clinical setting.

**Conclusion: **The PreMeFen study aims to evaluate whether two non-invasive analgesic methods are good enough for use in the non-physician ground ambulance setting and expand the armamentarium and accessibility of acute pain management.

**References**

1. Friesgaard KD, Vist GE, Hyldmo PK, Raatiniemi L, Kurola J, Larsen R, et al. Opioids for Treatment of Pre-hospital Acute Pain: A Systematic Review. Pain Ther. 2022 Mar;11(1):17–36.
2. Middleton PM, Simpson PM, Sinclair G, Dobbins TA, Math B, Bendall JC. Effectiveness of morphine, fentanyl, and methoxyflurane in the prehospital setting. Prehosp Emerg Care. 2010 Dec;14(4):439–47.

## P18 Investigating the impact of military traumatic brain injury on Quality-of-Life in the ADVANCE cohort of UK Afghanistan war veterans

### Daniele Ramsay^1,*^, David J. Sharp^2^, Neil S. N. Graham^2^

#### ^1^Imperial College School of Medicine, Imperial College London, London, UK. ^2^Department of Brain Sciences, Imperial College London, London, UK

##### *Correspondence: dsr2718@ic.ac.uk

***Scandinavian Journal of Trauma, Resuscitation and Emergency Medicine*** 2023, **31(1)**: P18

**Background: **Combat-related traumatic brain injury (CR-TBI) is a prominent feature of modern conflict, frequently due to blast exposure. Recently, there has been substantial research effort relating to the long-term associations of CR-TBI, including the armed services trauma rehabilitation outcome (ADVANCE) study [1]. Previous work has found CR-TBI affected veterans have worse outcomes when compared to veterans without injuries [2]. We aimed to clarify rates of CR-TBI and ascertain how this relates to health-related quality-of-life (HR-QoL) in servicemen with a history of combat trauma during the UK-Afghanistan campaign.

**Methods: **Past medical history data collected within ADVANCE were used to reach a preliminary CR-TBI rate, with determination of the mechanism of injury and stratification of severity according to the Mayo classification [3]. Study groups were formed according to whether a CR-TBI was experienced during the injury requiring medical evacuation, the ‘index injury’. Groups were then compared by demographics and HR-QoL measures. Summary HR-QoL scores, ranging from zero to one, were calculated combining values from the EQ-5D-5L questionnaire. Lower scores indicate a worse HR-QoL.

**Results: **A rate of all-severity CR-TBI during the index event, was 20.6% (119/579). Of these, 37 were moderate-severe (definite), 65 were mild (probable) and 17 were symptomatic (possible). Blasts caused 89.9% of these injuries (107/119). CR-TBI patients had worse HR-QoL outcomes compared to those exposed to battlefield trauma without CR-TBI, with scores 0.793 and 0.833 respectively (*p* = 0.006). There was a trend towards poorer outcomes in the moderate-severe (definite) group (0.770) compared with the mild (probable) group (0.798) and the symptomatic (possible) group (0.820), but this difference did not meet statistical significance (*p* = 0.656).

**Conclusion: **CR-TBI was frequently experienced in the ADVANCE cohort. CR-TBI exposure was strongly associated with poor HR-QoL. We did not demonstrate a significant dose–response effect of injury severity on outcome, reflecting the discrepancy between CR-TBI severity and HR-QoL outcomes.

**References**

1. Bennett AN, Dyball DM, Boos CJ, Fear NT, Schofield S, Bull AM, Cullinan P. Study protocol for a prospective, longitudinal cohort study investigating the medical and psychosocial outcomes of UK combat casualties from the Afghanistan war: the advance study. BMJ open. 2020 Oct 1;10(10):e037850.
2. Schiehser DM, Twamley EW, Liu L, Matevosyan A, Filoteo JV, Jak AJ, Orff HJ, Hanson KL, Sorg SF, Delano-Wood L. The relationship between postconcussive symptoms and quality of life in veterans with mild to moderate traumatic brain injury. Journal of Head Trauma Rehabilitation. 2015 Jul 1;30(4):E21-8.
3. Malec JF, Brown AW, Leibson CL, Flaada JT, Mandrekar JN, Diehl NN, Perkins PK. The mayo classification system for traumatic brain injury severity. Journal of neurotrauma. 2007 Sep 1;24(9):1417–24.

## P19 Characterising older trauma patients attended by prehospital doctor/paramedic teams: A national register-based study

### Hjalmar J Hansen^1^, Elisabeth Jeppesen^2^, Mostafa El Moumni^3^, Mathias Cuevas-Østrem^4*^

#### ^1^Faculty of Medicine, University of Groningen, Groningen, the Netherlands. ^2^Faculty of Health Sciences, University of Stavanger, Stavanger, Norway. ^3^Trauma Surgery, University Medical Center Groningen, Groningen, the Netherlands. ^4^Department of Research and Development, Norwegian Air Ambulance Foundation, Oslo, Norway

##### *Correspondence: mathias.cuevas-ostrem@norskluftambulanse.no

***Scandinavian Journal of Trauma, Resuscitation and Emergency Medicine*** 2023, **31(1)**: P19

**Background: **Severely injured patients over 65 years are at risk of not receiving advanced prehospital care [1, 2]. What characterises older trauma patients attended by doctor/paramedic teams is poorly described. The aim of this study was to compare characteristics of adult and older trauma patients attended by doctor/paramedic teams.

**Methods: **Data from the Norwegian Trauma Registry (2015–2020) was collected to identify patients who were attended by a doctor/paramedic team, age ≥ 16, and with a New Injury Severity Score (NISS) ≥ 9. Patients were stratified by age into Group 1 (G1, 16–64 years) and Group 2 (G2, ≥ 65 years). Differences in injury and management characteristics were assessed by using Chi-squared test and Mann–Whitney *U* test.

**Results: **We included 4126 patients in the analysis, of whom 959 (23%) in G2. The median NISS was 18 (IQR 12–29) for G1 and 22 (IQR 14–33) for G2 (*p* = 0.017). The median GCS was 15 (IQR 12–15) for G1 and 15 (IQR 11–15) for G2 (*p* = 0.004). Proportion GCS < 14 was 26.6% in G1 and 31.5% in G2 (*p* = 0.013). The mechanism of injury (MOI) was traffic-related in 49.9% in G1 and 41.6% in G2, high energy falls in 27.1% in G1 and 30.8% in G2, and low energy falls (LEFs) in 4.1% in G1 and 16.0% in G2 (*p* < 0.001). The likelihood of prehospital intubation (OR 1.10, 95% CI 0.91–1.32), chest decompression (OR 0.91, 95% CI 0.59–1.41), and transportation by air ambulance (OR 1.02, 95% CI 0.88–1.20) were similar between groups.

**Conclusion: **Older trauma patients attended by prehospital doctor/paramedic teams are more severely injured and have a lower GCS compared to their adult counterparts. High-energy injuries are the most common MOI in both groups, however, the proportion of LEFs is four times higher in the older population. Both groups are equally likely to receive advanced prehospital interventions and air ambulance transportation.

**References**

1. Cuevas-Østrem M, Wisborg T, Røise O, Jeppesen E. Differences in time-critical interventions and radiological examinations between adult and older trauma patients: A national register-based study. Journal of Trauma and Acute Care Surgery. 2022 Feb;93(4):503–12.
2. Eichinger M, Robb HDP, Scurr C, Tucker H, Heschl S, Peck G. Challenges in the PREHOSPITAL emergency management of geriatric trauma patients – a scoping review. Scand J Trauma Resusc Emerg Med. 2021

## P20 Emergency Department Resuscitative Thoracotomy for Traumatic Cardiac Arrest: outcomes of 18 cases conducted by London’s Air Ambulance

### S. Aziz^1,2,*^, R Greenhalgh^1,2^, A Whitehouse^1^, S Read^1^, E Foster^2^, C.L. Henry^1^, M Marsden^3,4^, R Lendrum^1,2^, D Lockey^1,2,3^, M.D. Christian^1^, Z.B. Perkins^1,2,3^

#### ^1^London’s Air Ambulance Charity, London, UK. ^2^The Royal London Hospital, Barts Health NHS Trust, London UK. ^3^Centre for Trauma Sciences, Queen Mary University of London, UK. ^4^Academic Department of Military Surgery and Trauma, Royal Centre for Defence Medicine, Birmingham, UK

##### *Correspondence: shadman.aziz@doctors.net.uk

***Scandinavian Journal of Trauma, Resuscitation and Emergency Medicine*** 2023, **31(1)**: P20

**Background: **Reported outcomes for resuscitative thoracotomy (RT) are better if the procedure is performed in-hospital compared to pre-hospital. This may be due to higher proportions of witnessed arrest, and better access to surgical equipment, expertise, blood products and lighting. We have previously reported 18% survival to hospital discharge for pre-hospital RT conducted by our service. The aim of this study was to assess survival in patients undergoing in-hospital RT by our teams.

**Methods: **A retrospective database review was conducted of all patients attended by London’s Air Ambulance (LAA). from the 1^st^ January 1999 to the 31^st^ December 2019. All RTs conducted by LAA clinicians in an Emergency Department (ED) were included. Outcomes were return of spontaneous circulation (ROSC), survived to hospital discharge, and survived with favourable neurological outcome (Cerebral Performance Category (CPC) score of ≤ 2).

**Results: **During the 20-year study period, 18 patients underwent ED RT by the LAA team. Median age was 23 (IQR 20–30) and 17 (95%) were male. In 9 (50%) cases, RT was performed on arrival at the destination hospital. In the other half, RT was performed at the referring hospital. Fifteen (83%) patients had penetrating injuries, the remaining 3 (17%) suffered blunt trauma. Six (33%) patients arrested due to cardiac tamponade, and 12 (67%) exsanguinated. One blunt trauma patient had tamponade. ROSC was achieved in 11 (61%) patients; and 3 (17%) survived to discharge, all with CPC score 1. Two patients (33%) with cardiac tamponade survived and one who had exsanguinated (8%). All survivors received RT within 1 min of arrest.

**Conclusion: **Patient outcomes are similar when RT is performed by the same team in pre-hospital or ED settings. For a witnessed traumatic cardiac arrest, pre-hospital teams that can perform RT should perform it immediately, even if a hospital is only a few minutes away.

## P21 The 7S strategies in managing COVID-19 pandemic in the tertiary care teaching hospital in Malaysia

### Afiq Mohd Nor^1,*^, Salleh Yahya^1^, Hafyzuddin Md Yusuf^2^

#### ^1^Emergency Medicine Department, University Malaya Medical Centre, Kuala Lumpur, Malaysia. ^2^Emergency Medicine Department, Faculty of Medicine, University of Malaya, Kuala Lumpur, Malaysia

##### *Correspondence: afiq@ummc.edu.my

***Scandinavian Journal of Trauma, Resuscitation and Emergency Medicine*** 2023, **31(1)**: P21

**Background: **The key challenge to any healthcare centre responding to a disaster, such as the COVID-19 pandemic, is its ability to rapidly address and react to the sudden influx of patients^1^ at both pre-hospital and in-hospital levels. The objective of this article is to report and share the experience of the physician and senior staff of University Malaya Medical Centre (UMMC), Kuala Lumpur, in preparing, responding and implementing management strategies for the COVID-19 pandemic, as well as highlighting how a tertiary teaching hospital copes with the challenges in handling the surge of COVID-19 cases during its peak time in Malaysia.

**Methods: **The master plan of COVID-19 surge capacity management was strengthened by the mix of seven fundamental components called 7 S’s: Space, Staff, Stuff, Supply, Support, System and Standard Operating Procedure (SOPs), which was overseen and discussed frequently during a regular special task force meeting.

**Results: **Elements discussed in this article comprise space (safe area and appropriate spaces with the capacity to serve a large number of patients), staff (qualified, well-trained personnel and sufficient to respond to needs), stuff (ensuring equipment and tools needed practically function and adequate), supply (plenty of medication stock and meals for patients and staff), support (financing, training, psychological), system (pre-hospital diversion and transfer, algorithm and workflow) and standard operating procedures, SOPs (active surveillance, risk-based assessment visa and infection prevention strategy). All these elements are built up based on the latest available scientific evidence, changes in the national guidelines and particular circumstances, and are recorded in the interim hospital protocol to be a reference for everyone.

**Conclusion: **Applying these 7S’s in the surge capacity management strategy can be helpful for teaching hospitals to respond to sudden influx situations in any disaster event, and the lessons learned from this approach should be shared as they will guide others in pre- and post-pandemic preparations.

**Reference**

1. Hick JL, Barbera JA, Kelen GD. Refining Surge Capacity: Conventional, Contingency, and Crisis Capacity. Disaster Medicine and Public Health Preparedness. Cambridge University Press; 2009;3(S1): S59–S67.

## P22 A one-year review of prehospital point-of-care ultrasound at London’s Air Ambulance.

### Salman Naeem^1,*^, Thomas Hirst^2^, Matthew Mak^1^, Michael, Palmer^2^, Jonas Schlautmann^1^, Thomas Hurst^1^, Daniel Nevin^1^

#### ^1^London’s Air Ambulance Charity, London, United Kingdom. ^2^Adult Critical Care Transfer Service Cymru, Wales, United Kingdom

##### *Correspondence: salman.naeem@nhs.net

***Scandinavian Journal of Trauma, Resuscitation and Emergency Medicine*** 2023, **31(1)**: P22

**Introduction: **Point-of-care ultrasound (PoCUS) has seen an increase in use in prehospital environment [1,2]. It was introduced at the London’s Air Ambulance (LAA) service in September 2021.

**Methods: **This is a retrospective service evaluation of the PoCUS program at LAA from 1^st^ September 2021 to 1st September 2022. Scans were performed by clinicians who underwent training within the service. Images were automatically uploaded to google drive after scanning. All scans were periodically reviewed by a group of experts in PoCUS. Both clinicians and reviewers documented their findings in a standardised online form on REDCap. Data were exported to Microsoft EXCEL for analysis. Percentages were calculated for continuous data and kappa was calculated between reviewers and clinicians for each modality.

**Results: **A total of 454 scans were included. After removing 10 duplicates, 444 were analysed. The population scanned were predominantly male (86%) with 10% paediatric patients. Majority (93%) of scans were done for trauma, of which 8% of patients were in cardiac arrest. 46% and 69% had suspected chest and abdominal injuries respectively. Reviewers deemed 84% of scans of adequate quality with kappa of 0.29. A total of 181 subcostal scans were performed for pericardial effusion, 277 thoracic scans for pneumothorax, 144 for pleural effusion, and 199 right upper quadrant scans for free fluid in peritoneum with kappa of 0.64, 0.7, 0.63 and 0.6 respectively. Clinical impact of PoCUS was recorded in 419 scans where it confirmed diagnosis in 73% encounters while it ruled out pathology in 25% of them. Issues with ultrasound machine, patient, environment, and operator were recorded in 13.5%, 15%, 13% and 5% of encounters respectively.

**Discussion and conclusion: **It is feasible to perform good quality PoCUS in prehospital environment with high inter-operator reliability. It is valuable to clinicians to rule-in or rule-out pathologies and guides in decision making.

**Acknowledgments**

We would like to acknowledge the contributions of the LAA POCUS Group which includes Richard Muswell, Michael Christian, Angus Perks, Behnaz Mahmoodian and Adrian Wong.

**References**

1. Taylor J, McLaughlin K, McRae A, et al*.* Use of prehospital ultrasound in North America: a survey of emergency medical services medical directors. BMC Emerg Med 14, 6 (2014). https://doi.org/10.1186/1471-227X-14-6
2. El Sayed MJ, Zaghrini E. Prehospital emergency ultrasound: a review of current clinical applications, challenges, and future implications. Emerg Med Int. 2013;2013:531,674. https://doi.org/10.1155/2013/531674. Epub 2013 Sep 19. PMID: 24,171,113; PMCID: PMC3792527.

## P23 Prehospital point-of-care ultrasound for diagnosis of pericardial effusion: A systematic review and meta-analysis

### Salman Naeem^1,*^, Edwin Li Ping Wah-Pun Sin^2^, Joyce Cheng^3^, Amir Alzarrad^4^, Serena Rovida^5^

#### ^1^Emergency Department, East Kent Hospitals University Foundation Trust, Ashford, United Kingdom. ^2^Intensive care, West Suffolk NHS Foundation Trust, St Bury Edmunds, United Kingdom. ^3^Internal medicine, Cambridge University NHS Foundation Trust, Cambridge, United Kingdom. ^4^Accident & Emergency, Barts Health NHS Trust, London, United Kingdom. ^5^Intensive care Unit, St Georges’ Hospital, London, United Kingdom

##### *Correspondence: salman.naeem@nhs.net

***Scandinavian Journal of Trauma, Resuscitation and Emergency Medicine*** 2023, **31(1)**: P23

**Background: **Pericardial effusion is difficult to diagnose on clinical examination in prehospital environment and cardiac ultrasound is the investigation of choice. It is unclear how accurate is prehospital point-of-care ultrasound (PoCUS) for diagnosis of pericardial effusion [1,2]. The aim of this systematic review & meta-analysis was to assess the accuracy of prehospital ultrasound for diagnosis of pericardial effusion.

**Methods: **We conducted a systematic literature search on Pubmed, Medline, SCOPUS, Cochrane database of systematic review, EMBASE and references of included studies between 1988 to 2022 on all patients having prehospital ultrasound for diagnosis of pericardial effusion. We then performed a risk of bias analysis and descriptive data analysis. Data from diagnostic studies was extracted and 2 × 2 table was constructed. The systematic review & meta-analysis was conducted in line with Preferred Reporting Items for Systematic reviews and Meta-Analyses (PRISMA) statement and registered with PROSPERO (registration number CRD42022354618).

**Results: **We identified 437 citations and included 17 articles in the final review. A majority of the studies included were case reports and case series (76%) while four retrospective and prospective diagnostic studies were identified. There was a high risk of bias in the studies included. A total of 665 cardiac scanning encounters were included with a combined sensitivity, specificity, PPV and NPV of 54%, 99.8%, 92.3% and 98.4%.

**Discussion and conclusion: **There is scarcity of literature on the diagnostic accuracy of prehospital PoCUS for pericardial effusion. A majority of the studies included were case reports and case series with high risk of bias. The four studies included had small sample sizes and none had the above question as the primary outcome. This systematic review and meta-analysis highlights that prehospital PoCUS may be feasible to either rule in or rule out pericardial effusion however, further studies are required to assess this outcome.

**References**

1. Partyka, C., Coggins, A., Bliss, J. et al. A multicenter evaluation of the accuracy of prehospital eFAST by a physician-staffed helicopter emergency medical service. Emerg Radiol 29, 299–306 (2022). https://doi.org/10.1007/s10140-021-02002-4
2. Press GM, Miller SK, Hassan IA, Alade KH, Camp E, Junco DD, Holcomb JB. Prospective evaluation of prehospital trauma ultrasound during aeromedical transport. J Emerg Med. 2014 Dec;47(6):638–45. https://doi.org/10.1016/j.jemermed.2014.07.056. Epub 2014 Oct 1. PMID: 25,281,177.

## P24 Management of Major Haemorrhage in those who decline blood products: a guideline

### Carl Groves^1,*^, Laura Conway^1^, Laura May^1^

#### ^1^Anaesthetics Department, University Hospital, Coventry, UK

##### *Correspondence: carl.groves@uhcw.nhs.uk

***Scandinavian Journal of Trauma, Resuscitation and Emergency Medicine*** 2023, **31(1)**: P24

University Hospital, Coventry is a major trauma centre serving a large proportion of the Midlands and the surrounding areas. It managed 1607 trauma cases in 2021(1); many of these involving major haemorrhage. The use of a major haemorrhage protocol (MHP) is one of the management pillars of such patients allowing rapid access to large volumes of blood products (2).

More recently we have managed several cases where the patient refuses blood products. This can be on religious grounds such as in the Jehovah’s Witness or Rastafarian faiths. In addition there are patients who refuse blood products due to safety concerns (3). Working in collaboration with the local haematology and legal departments as well as the Jehovah’s witness hospital liaison committee we have developed a guideline to assist clinicians when managing massive haemorrhage in major trauma in those who refuse blood.

Our aim was to give clinicians more clarity in complex, high pressure and time critical situations. The overriding principle at all stages is discussion with the patient, when they have capacity, to find which products they consent to. This must be regularly reviewed at key decision points with the risks and benefits of transfusion and declining it being covered in an open, non-judgmental way. Patients always have the right to alter their decisions. Our guideline considers several different scenarios the clinician may be presented with in a trauma patient who declines blood products. We have produced a flow diagram if a patient has an advanced directive and to confirm its validity; how to manage the patient if they lack capacity and who to contact in any eventuality. We have also provided a quick reference as to the possible alternatives and where they can be quickly accessed.

**References**

1. University Hospital of Coventry & Warwickshire Hospital Details: Trauma Audit & Research Network; 2021 [Available from: https://www.tarn.ac.uk/Content.aspx?ca=15&c=2897&hid=8810.
2. Spahn DR, Bouillon B, Cerny V, Duranteau J, Filipescu D, Hunt BJ, et al. The European guideline on management of major bleeding and coagulopathy following trauma: fifth edition. Crit Care. 2019;23(1):98.
3. Caring for patients who refuse blood—a guide to good practice. England: Royal College of Surgeons of England; 2016.

## P25 A 12-month retrospective review of the incidence of anticoagulant and antiplatelet use in patients with significant head injuries presenting to a major trauma centre

### Rory Carroll^1,*^, Isabel Fox^1^, Tharanya Thiruchelvam^1^, Anthony Hudson^2^, Harriet Tucker^2^

#### ^1^Faculty of Health, University of Plymouth, Plymouth, United Kingdom. ^2^Accident and Emergency Department, St George’s University Hospitals NHS Foundation Trust, London, United Kingdom

##### *Correspondence: is18954@bristol.ac.uk

***Scandinavian Journal of Trauma, Resuscitation and Emergency Medicine*** 2023, **31(1)**: P25

**Background: **Each year 1.4 million patients attend emergency departments with head injuries in England and Wales [1]. Anticoagulant and antiplatelet use is associated with increased risk of intracranial haemorrhage following head injury and challenges resulting from an aging population suggest this burden is only increasing [2]. Targeted management of specific anticoagulants is expensive and it is unclear how to best manage patients on antiplatelets. Although, the CRASH-3 trial has indicated a benefit of early tranexamic acid (TXA) administration in mild-moderate head injury [3].

**Methods: **A retrospective review of all patients admitted to a UK Major Trauma Centre (MTC) with significant head injury over a 12-month period, between 01/01/2021 and 31/12/2021. Trauma Audit and Research Network (TARN) data was extracted and compared to individual electronic date records. Retrospective analysis included anticoagulant and/or antiplatelet use, reversal agent use and when administered, time to TXA administration.

**Results: **468 patients presented with a significant head injury. 67.9% were male with a mean age of 61.2 years. 14.3% were anticoagulated at time of injury, with large variability in agents used. 17.7% were on antiplatelets, of which 25.3% were on dual antiplatelet therapy. TARN data significantly underreported the use of both anticoagulants and antiplatelets in this population. Only 33.1% received TXA and the mean time from injury to administration was 1 h 39 min.

**Conclusion: **Large numbers of significantly head injured patients are on anticoagulants or antiplatelets. Clinicians often administered non-specific reversal agents: this may be due to the wide variability of anticoagulants encountered, and costs. Antiplatelets in head injury present a significant clinical challenge due to lack of evidence on how to best manage them. Further research is required to improve patient outcomes. Strategies to improve early recognition of head injury and encourage administration of TXA may also provide clinical benefits.

**References**

1. National Institute for Health and Care Excellence (NICE). Head injury: quality standards and indicators briefing paper. [2014] Available from: https://www.nice.org.uk/guidance/qs74/documents/head-injury-briefing-paper2 Accessed 20/10/2022.
2. Van den Brand C, Tolido T, Rambach A, Hunink M, Patka P, Jellema K. Systematic review and meta-analysis: is pre-injury antiplatelet therapy associated with traumatic intracranial hemorrhage? J Neurotrauma 2017; 34(1):1–7. doi:10.1089/neu.2015.4393
3. Roberts I, Shakur-Still H, Aeron-Thomas A, Belli A, Brenner A, Anwar Chaudary M et al*.* Effect of tranexamic acid on death, disability, vascular occlusive events and other morbidities in patients with acute traumatic brain injury (CRASH-3): a randomised, placebo-controlled trial. The Lancet. 2019; 394(10,210):1713–1723

## P26 Who survives out-of-hospital cardiac arrests attended by pre-hospital critical care teams in wales?

### Elliot Phillips^1^, Thomas Archer^2^, David Rawlinson^2^

#### ^1^School of Medicine, Cardiff University, Cardiff, UK. ^2^Emergency Medical Retrieval & Transfer Service, Dafen, Carmarthenshire, UK

##### *Correspondence: PhillipsEJ1@cardiff.ac.uk

***Scandinavian Journal of Trauma, Resuscitation and Emergency Medicine*** 2023, **31(1)**: P26

**Background: **The Emergency Medical Retrieval and Transfer Service (EMRTS) are Wales’ pre-hospital critical care organisation. Out-of-hospital cardiac arrests (OHCA) comprise 21% of the calls EMRTS attend annually. Despite advancements in resuscitation science and interventions from the Welsh Government, mortality from OHCA in Wales remains one of the worst in Western Europe at 4.6% [1].

**Method: **A retrospective review of EMRTS’ database was performed to identify OHCA attended between April 2019 and March 2020. 394 incidents were identified after exclusions. Patients conveyed to hospital with an NHS number recorded were analysed (n = 92). These patients were cross-referenced with national databases to investigate 9 potential survivor characteristics. Pre-arrest, intra-arrest, post-arrest and Utstein criteria characteristics [2] were included. Socioeconomic deprivation of patients was identified using Welsh Index of Multiple Deprivation.

**Results: **Twenty-five patients (27.1%) analysed were alive at 30 days. This represents 8.4% of all OHCA EMRTS attended in this period. Survival was associated with lower socioeconomic deprivation (*p* = 0.06), the presence of bystander CPR, witnessed arrests, and an initial shockable initial rhythm. Response time by EMRTS did not affect survival (*p* = 0.72).

**Conclusion: **Despite critical care interventions, mortality from OHCA attended by EMRTS is as low as Wales’ nationwide survival rate. In a country with high socioeconomic deprivation, this study has shown it to be a significant factor behind OHCA survival. Bystander CPR, shockable rhythm and witnessed arrests remain positive prognostic factors. Further analysis using the planned OHCA registry for Wales will allow a larger sample to be analysed.

**References**

1. Welsh Government. Lifesaving programme given cash boost to keep going by Welsh Government. 2021. https://media.service.gov.wales/news/lifesaving-programme-given-cash-boost-to-keep-going-by-welsh-government. Accessed 5 Aug 2022
2. Perkins G, Jacobs I, Nadkarni V, Berg RA, Bhanji F, Biarent D, et al. Cardiac Arrest and Cardiopulmonary Resuscitation Outcome Reports: Update of the Utstein Resuscitation Registry Templates for Out-of-Hospital Cardiac Arrest. Circulation. 2015;132(13):1286–300. https://doi.org/10.1161/CIR.0000000000000144

## P27 Delivering Extracorporeal Cardiopulmonary Resuscitation across a rural ambulance service footprint; a retrospective cohort feasibility study

### Frederick Stourton^1,*^, Andrew Heavyside^2^, Aidan Joyce^3^, Matt Thomas^4^

#### ^1^Worthing Hospital, University Hospitals Sussex NHS Foundation Trust. ^2^University Hospital Southampton NHS Foundation Trust. ^3^Great Western Air Ambulance Charity.

##### *Correspondence: freddiestourton@doctors.org.uk

***Scandinavian Journal of Trauma, Resuscitation and Emergency Medicine*** 2023, **31(1)**: P27

**Background: **Pre-hospital Extracorporeal Cardiopulmonary Resuscitation (ECPR) has been shown to be feasible in large urban centres but there is minimal reported use in more remote areas. Our aim was to establish how many patients in a rural ambulance footprint might benefit from ECPR, and the feasibility of delivering it.

**Methods: **All cardiac arrests in the South West Ambulance Service Foundation Trust (SWASFT) registry from a 1 year period were analysed for potential suitability for ECPR according to the criteria used for the SUB30 trial. Response times for each case were then generated according to one of four scenarios; (a) existing Helicopter Emergency Medical Service (HEMS) services, (b) 24-h HEMS services, (c) a new ‘working hours’ ground vehicle-based service from specialist centres, (d) 24-h ground vehicle-based service.

**Results: **Of the 3711 cardiac arrests in the 12-month period, 173 met the eligibility criteria and may have benefited from ECPR. The ground vehicle service would reach 8% of patients (working hours) within 30 min, rising to 23% when operating over 24 h. Existing HEMS resources would reach 53% of cases within 30 min, rising to 69% if operating 24/7.

**Discussion: **This study suggests that there are patients who could benefit from ECPR outside major urban centres, and that HEMS would be key to delivering it given the distances required. We acknowledge the organisational, resource and ethical considerations involved in delivering such an intervention.

**Acknowledgements**

The authors are grateful to Dr Sarah Black and SWASFT for access to the cardiac arrest registry.

## P28 Success rate of prehospital emergency front of neck access (FONA): a systematic review and meta-analysis

### Sarah Morton^1,*^, Pascale Avery^2^, Justin Kua^3^, Matt O’Meara^1^

#### ^1^Essex and Herts Air Ambulance, Colchester, United Kingdom. ^2^Emergency Retrieval and Transfer Service (EMRTS) Wales Air Ambulance, Dafen, Wales, United Kingdom. ^3^Research Department of Targeted Intervention, University College London, London, United Kingdom

##### *Correspondence: sarah.morton@doctors.org.uk

***Scandinavian Journal of Trauma, Resuscitation and Emergency Medicine*** 2023, **31(1)**: P28

**Background: **Front of neck access (FONA) is an emergency procedure which may be used as a last resort to achieve a patent airway in the prehospital environment. Various techniques exist including the surgical technique outlined by the UK Difficult Airway Society in a ‘can’t intubate, can’t oxygenate’ scenario. This technique has been recommended as the first line prehospital technique since 2017. This systematic review and meta-analysis aims to evaluate the incidence and success rate of FONA in the prehospital setting, including changes since 2017.

**Method: **A systematic literature search was performed from inception to July 2022 to identify studies in patients of any age undergoing prehospital FONA and data extracted. Meta-analysis was used to derive pooled success rates. Methodological quality of included studies was interpreted using the Cochrane risk of bias tool, and rated using the Grading of Recommendations Assessment, Development and Evaluation (GRADE) approach.

**Results: **From 909 studies, 69 studies were included (33 low quality; 36 very low quality) with 3292 prehospital FONA attempts described (1229 available for data analysis). Crude success rates ranged from 0 to 100%, with a median of 100%, increasing from 99.2% pre-2017 to 100% post-2017. The random-effects meta-analysis showed an estimated overall pooled FONA success rate of 88% (95% Confidence Interval (95% CI): 85%–91%). Surgical techniques had the highest success rate at a median of 100% (pooled effect 92%; 95% CI: 88%–95%) versus 50% for needle techniques (pooled effect 52%; 95% CI: 28%–76%). Physician-led prehospital teams had the highest success rate at a median of 100% (pooled effect 89%; 95% CI: 83%–93%). Paediatric cases had a pooled success rate of 74% (95% CI: 56–87%).

**Conclusion: **Despite being a relatively rare procedure in the prehospital setting, the success rate for FONA is high. A surgical technique for FONA appears more successful and supports the existing UK prehospital guidelines.

## P29 HEMS provide critical interventions at the scene of firearms incidents in a semi-rural region of the UK

### Rob Loveless^1^, Anthony Hudson^1,2^, Jo Griggs^1^, Richard Lyon^1,*^

#### ^1^Air Ambulance Charity Kent Surrey Sussex, Redhill Aerodrome, Surrey. ^2^Department of Emergency Medicine, St Georges NHS Foundation Trust, London

##### *Correspondence: RichardLyon@aakss.org.uk

***Scandinavian Journal of Trauma, Resuscitation and Emergency Medicine*** 2023, **31(1)**: P29

**Introduction: **Nationally, firearms incident rates have been consistent since 2012 [1], representing less than 1% of offences reported to Police [1]. However, the incidence of firearms incidents in the South-East has risen^1^. Air Ambulance Kent Surrey Sussex is a Helicopter Emergency Medical Service (HEMS) providing pre-hospital care to South-East England. The service covers a mixed rural and urban environment, operating 24/7. We present our experience of attending firearms incidents.

**Methods: **In this retrospective analysis we reviewed all cases recorded in our electronic patient database between 1/7/2013 and 3/8/2022 involving firearms. We provide a descriptive analysis of data regarding injury pattern, weapon, interventions, triage and survival to hospital.

**Results: **In 9 years we attended 47 firearms incidents. 2 patients were excluded (one treated by another HEMS service, one uninjured), and the remaining 45 cases were classified by transport method (Aircraft Carry, Ground Escort, Ground Assist, or non-survivors to hospital). 32 (70%) either required HEMS escort to hospital or did not survive. The remaining 13 (30%) did not require HEMS escort. A higher proportion of patients who survived to hospital sustained head and neck injuries, and 40% required RSI for airway protection. Chest and abdominal injuries were more common in the non-survivor group. 80% in traumatic cardiac arrest received HOT algorithm (intubation, thoracostomies and consideration of blood transfusion), and 2 patients underwent resuscitative thoracotomy. No thoracotomy cases survived to hospital.

**Discussion: **Injuries amenable to treatment in penetrating trauma are those with compressible haemorrhage and non-compressible haemorrhage is associated with non-survival to hospital. Blood transfusion was rarely indicated, since our Standard Operating Procedure aims to be restrictive in penetrating trauma, facilitating permissive hypotension [2]. Minimising scene time is essential, but HEMS offer significant interventions of haemorrhage control and RSI for airway protection in those with penetrating neck trauma, who would otherwise lose airway patency prior to hospital.

**References**

1. Allen G, Harding M. Firearm Crime Statistics: England and Wales. *Office for National* Statistics, 2021. Available online at: https://researchbriefings.files.parliament.uk/documents/CBP-7654/CBP-7654.pdf
2. Bickell et al. Immediate versus delayed fluid resuscitation for hypotensive patients with penetrating torso injuries. *NEJM,* 1994*.* **331:**1105–1109

## P30 Evaluating pre-hospital time and factors associated with delay for injured patients during trauma-related mass casualty incidents: a systematic review

### Fayez Alruqi^1,*^, Elom K. Aglago^2^, Elaine Cole^1^, Karim Brohi.^1^

#### ^1^Centre for Trauma Sciences, Blizard Institute, Queen Mary University of London, London, UK. ^2^Department of Epidemiology and Biostatistics, School of Public Health, Imperial College London, London, UK

##### *Correspondence: F.alruqi@qmul.ac.uk

***Scandinavian Journal of Trauma, Resuscitation and Emergency Medicine*** 2023, **31(1)**: P30

**Background: **Mass casualty incidents (MCIs) have risen globally over the last two decades, with over two million associated fatalities. [1] Recent reports suggest that critically injured patients experience delays in being transported to major trauma centres during an MCI and extended pre-hospital times (PHTs) are associated with increased mortality rate. [2] The primary aim of this systematic review was to evaluate PHTs for injured patients during trauma MCIs. The secondary aim was to evaluate factors associated with pre-hospital delays within trauma MCIs.

**Methods: **The systematic review protocol was registered in PROSPERO (CRD42022288580). Studies of any methodology reporting PHTs for triaged patients during trauma-related MCIs were eligible for inclusion. Web of Science, CINAHL, MEDLINE, and EMBASE were searched for evidence. Newcastle–Ottawa Scale and Joanna Briggs Institute were used to assess quality and risk of bias. A narrative synthesis was performed according to Cochrane guidance [3].

**Results: **Of the 2024 publications identified from the initial search, 12 papers met the eligibility criteria. Six observational cohort studies and six case reports reported a diverse range of MCIs involving 2718 trauma patients. Of these 93.30%, (2536) were formally triaged at incident scenes. PHTs varied widely across incidents, ranging from a median of 35 min (balcony collapse) to 8 h 8 min (remote bus rollover), whilst the majority reported PHTs of two hours or longer. Factors associated with delayed PHT included incident location and geography, scene safety, and poor decision-making in MCI responses. Study quality was generally low, with a low to moderate risk of bias.

**Conclusion: **MCIs within this review were heterogeneous in nature, and PHTs ranged widely, with many delays exceeding two hours. Further investigation is merited into the modifiable factors associated with delays, such as EMS providers’ decision-making.

**References**

1. Centre for Research on the Epidemiology of Disasters and UN Office for Disaster Risk Reduction. Human cost of disasters: an overview of the last 20 years 2000–2019. Emergency Events Database (EM-DAT) Centre for Research on the Epidemiology of Disasters, 2020: 1–17.
2. Kim J, Kim CH, Shin SD, Park JO. Prehospital Response Time Delays for Emergency Patients in Events of Concurrent Mass Casualty Incidents. Disaster Med Public Health Prep. 2018;12(1):94–100.
3. Ryan R; Cochrane Consumers and Communication Review Group. Cochrane Consumers and Communication Review Group: data synthesis and analysis. 2013 Jun [cited 2021 Dec 6]. Available from: https://cccrg.cochrane.org/sites/cccrg.cochrane.org/files/public/uploads/AnalysisRestyled.pdf.

## P31 Variability in approach to informing the relatives of non-surviving participants in cardiac arrest research: a questionnaire study

### Abigail Dove^1, *^, Laura Pointeer^1^, Keith Couper^1,2^, Gavin D. Perkins^1,2^, Helen Pocock^1,3^

#### ^1^University of Warwick, Coventry, United Kingdom. ^2^University Hospitals Birmingham NHS Foundation Trust, Birmingham, United Kingdom. ^3^South Central Ambulance Service NHS Foundation Trust, Winchester, United Kingdom

##### *Correspondence: Abigail.Dove@warwick.ac.uk

***Scandinavian Journal of Trauma, Resuscitation and Emergency Medicine*** 2023, **31(1)**: P31

**Purpose of the study: **Cardiac arrest trial participants are often enrolled under a waiver of consent. We conducted a survey study to describe what approaches have been adopted to informing relatives of trial participants who did not survive about clinical trial participation.

**Materials and methods: **For this survey study, we identified participants by searching three clinical trial registries for human out- of-hospital and in-hospital cardiac arrest randomised controlled trials conducted since 2010 that evaluated intra-arrest interventions. The named contact listed on the trial registry entry was invited to complete the survey on behalf of their study. We developed the survey in collaboration with experienced critical care researchers to ensure content validity. Closed and open questions were included, and piloting was conducted to ensure appropriateness of response options. Closed response answers were analysed using descriptive statistics whilst open responses were coded to identify themes. Ethical approval was granted by the University of Warwick Biomedical and Scientific Research Ethics Committee.

**Results: **Of 36 identified study contacts, we received 15 responses (response rate = 42%) from six countries across North America and Europe. In five studies (31%), relatives were actively and directly informed of relatives’ participation- this was done by telephone (n = 2, 40%), letter (n = 1, 20%), by the clinician at scene (n = 1, 20%), or a researcher post event (n = 1, 20%). In seven studies (47%), a passive method of informing was used, whereby posters were displayed where relatives were likely to see them. In the remaining four studies (27%), relatives were not informed about enrolment. Across all studies, the trial contact agreed or strongly agreed that they had adopted the best approach in relation to providing information to relatives.

**Conclusions: **There is variability across studies in the way that information about trial participation is provided to relatives of non- survivors.

## P32 Occupational therapist-led assessment of functional ability, cognition, mood, fatigue and quality of life in out of hospital cardiac arrest survivors

### Méadbh Keenan^1,*^, Monique Debsie-Smith^1^, Margie Crouch^1^, Anthony Bastin^1^, Nikos Gorgoraptis^1,2^

#### ^1^Barts Heart Centre, St Bartholomew’s Hospital, Barts Health NHS Trust, London, UK. ^2^Preventive Neurology Unit, Wolfson Institute of Preventative Medicine, Queen Mary University of London, UK

##### *Correspondence: meadbh.keenan@nhs.net

***Scandinavian Journal of Trauma, Resuscitation and Emergency Medicine*** 2023, **31(1)**: P32

**Background: **Following Out of Hospital Cardiac Arrest (OHCA) survivors may experience neurocognitive problems including cognitive impairment and fatigue which can affect functional ability and quality of life, but can be missed if not screened for. It is not clear what Occupational Therapist (OT)-led assessments are suitable for screening in the acute setting and virtual follow-up

**Materials and methods: **OHCA inpatients were assessed using RUDAS, AMTS10 or FreeCog, in function, or remotely post discharge using TeleCog. Individuals with cognitive impairment were offered further assessment in follow-up clinic within 3 months using TeleCog. Fatigue was examined using VAFS and health-related quality of life using EQ-5D-5L. Data was collected between November 2021 and August 2022.

**Results: **77 OHCA patients (median age 58 years (range: 23–83), 71 male, 6 female) were assessed; 54% in function and 93% using a standardised cognitive assessment. 50% of patients assessed using the RUDAS (N = 32), FreeCog (N = 40) or TeleCog (N = 18) evidenced impaired memory. 21% of those assessed in function showed impairment of executive function. Patients reported fatigue (VAFS; N = 20; mean: 5/10; SD: 2) and impaired quality of life EQ-5D-5L (N = 44; mean: 58/100; SD: 26).

**Conclusion: **OT-led assessment using FreeCog, functional assessment, VAFS and EQ-5D-5L is feasible in a tertiary centre treating a high volume of OHCA patients. Advantages of FreeCog versus AMTS10 and RUDAS include executive function questions and availability of a virtual version. Impairments were noted in a significant proportion of patients whose needs may have not been identified otherwise.

**References**

1. Moulaert V, Verbunt J, van Heugten C, Wade D. Cognitive impairments in survivors of out-of-hospital cardiac arrest: a systematic review. Resuscitation. 2009; 80(3): 297–305.
2. Nolan, J. European Resuscitation Council Guidelines for Resuscitation 2021. Resuscitation. 2021..
3. The National Confidential Enquiry into Patient Outcome and Death. Time Matters. 2021. London.

## P33 Staff-perceived barriers to trauma team performance in a low-volume major trauma centre: a questionnaire study

### Hephzibah Adegoke^1^, Rachel Barker^1^, Remona Karim^1^, Anna Kun^1^, Hannah McIntyre^1^, Elgan Manton-Roseblade^1^, Sarah Tan^1^, Nicole D Vance^1^, Zixin G Yang^1^, Yujia Zhu^1^, Giulio Allori^2^, Barry Schyma^3,*^

#### ^1^University of Edinburgh Medical School, Edinburgh, UK. ^2^Dept of Anaesthesia and Critical Care, Royal Infirmary of Edinburgh, Edinburgh, UK. ^3^Centre for Trauma Sciences, Queen Mary University of London, Blizard Institute, London, UK

##### *Correspondence: b.schyma@qmul.ac.uk

***Scandinavian Journal of Trauma, Resuscitation and Emergency Medicine*** 2023, **31(1)**: P33

**Background: **Cohesive trauma teams are vital to time-critical management of severely injured patients [1]. Trauma team performance research is often focused on high-volume trauma centres which may not translate to low-volume centres where infrequency can lead to inexperienced and unfamiliar teams. We aimed to investigate factors influencing trauma team performance in a low-volume major trauma centre (MTC), identify barriers and generate suggestions for improvement.

**Materials and methods: **An online questionnaire was distributed to trauma team members at the Royal Infirmary of Edinburgh, a low-volume MTC. Quantitative and qualitative data was generated which investigated staff performance and contributing factors. We focused on perceived personal confidence, team familiarity and training. Suggestions for improving the trauma team experience were collected. Data was analysed using Excel and NVivo.

**Results: **115 multi-disciplinary responses met inclusion criteria. 31% didn’t feel confident when attending trauma calls and 44% felt unable to perform at their best. Only 55% felt confident they were familiar with abilities and skill sets of others. Unfamiliarity with team members was perceived to negatively impact personal performance (37%) and was associated with barriers to communication. Low rates of role induction (40%) and educational action card use (34%) negatively impacted perceived personal performance. Only 34% of staff undertook any recent trauma training and only 17% had multi-disciplinary team training. Additional barriers included understaffing and lack of major trauma experience Multi-disciplinary simulation training, technical skill training and a greater attention to introductions and debriefs were felt to address familiarity and education issues.

**Conclusion: **Multifactorial challenges in delivering team-based trauma care in a low-volume MTC may result in low individual and team confidence, and suboptimal performance. Patients require optimal care despite centre volume and increased multi-disciplinary simulation training may be useful in achieving this. Further work in low-volume MTCs is required to understand impacts on patient outcomes.

**Reference**

1. Speck R, Jones G, Barg F, McCunn M. Team composition and perceived roles of team members in the trauma bay. Journal of Trauma Nursing [Internet]. 2012 [cited 4 October 2022];19(3):133–138. Available from: https://pubmed.ncbi.nlm.nih.gov/22955707 doi:10.1097/JTN.0b013e318261d273

## P34 Derivation of ultra-short term heart rate variability using carotid-femoral arterial waveforms in injured British military servicemen

### Rabeea Maqsood^1,*^, Alexander N. Bennett^2^, Christopher J. Boos^3^

#### ^1^Faculty of Health and Social Sciences, Bournemouth University, Bournemouth, Dorset, UK. ^2^Academic Department of Military Rehabilitation, Defence Medical Rehabilitation Centre, Loughborough, Leicestershire, UK. ^3^Department of Cardiology, University Hospital Dorset, NHS Trust, Poole, Dorset, UK

##### *Correspondence: rmaqsood@bournemouth.ac.uk

***Scandinavian Journal of Trauma, Resuscitation and Emergency Medicine*** 2023, **31(1)**: P34

**Background: **Heart rate variability (HRV) has evolved as a useful indirect measure of injury severity and adverse outcomes following traumatic injury. The comparative utility of proximal versus distal arterial sites to determine the cardiac inter-beat interval (IBI) for ultra-short term heart rate variability (HRV_UST_) measurement, following traumatic injury, has not been investigated and was the focus of this study.

**Method: **This was a cross-sectional observational study of 50 non-acutely injured military servicemen (aged > 18 years) from the ArmeD serVices trAuma and rehabilitatioN outComE (ADVANCE) study, UK. Cardiac IBIs were obtained in triplicate over 14-s recording of carotid-femoral waveforms obtained during pulse wave velocity measurement (Vicorder device). Arterial waveform data were exported to Kubios HRV software for analysis and reported as the root mean square of the successive differences between consecutive heart beats (RMSSD). For paired comparative analysis, Spearman correlation, Wilcoxon signed-rank test and Coefficient of Variance were computed with significance level set at *p* < 0.05. The inter-rater reliability between two blinded examiners were calculated using the Intra-class Correlation Coefficient (ICC) and Bland–Altman plots.

**Results: **The mean age of participants was 34.06 ± 4.88 (range: 23–44) years. The femoral waveform offered a more reliable signal than that of the carotid artery (Fisher’s Exact test; *p* < 0.0001). Carotid-derived RMSSD was higher (z = 4.468, *p* < 0.0001) and moderately correlated with that of the femoral artery (r = 0.82, *p* < 0.0001). There was a strong inter-rater correlation [ICC 0.99 (95%CI: 0.994–0.997)] and agreement in femoral-derived RMSSD scores (mean difference: 0.55 ms; 95% limit of agreement −7.21 to 8.32 ms).

**Conclusion: **This is the first study to compare proximal versus distal large arterial waveforms to measure HRV_UST_ in an injured military population and demonstrated the superiority of the femoral arterial signal. Our results offer the translational potential to utilise the femoral arterial signal to enhance clinical risk stratification following traumatic injury.

**Acknowledgements**

This study is a part of RM’s PhD Studentship which is jointly funded by Bournemouth University and the ADVANCE charity, UK. We would like to additionally acknowledge the support of Professor Paul Cullinan, Professor Nicola Fear, Professor Anthony Bull, Susie Schofield and Emma Coady from the ADVANCE study.

## P35 Management and outcomes of extremity vascular injury at a UK Major Trauma Centre

### Inova S. W. Lee^1,*^, Zane Perkins^2^, Ross Davenport^1^

#### ^1^Centre for Trauma Sciences, Queen Mary University of London, London, UK. ^2^The Royal London Hospital, Barts Health NHS Trust, London, UK

##### *Correspondence: inova.lee@nhs.net

***Scandinavian Journal of Trauma, Resuscitation and Emergency Medicine*** 2023, **31(1)**: P35

**Background: **Extremity vascular injury (EVI) can cause ischaemia or haemorrhage and result in limb loss or mortality. Current literature varies over the surgical management for achieving optimal outcomes. We examined our treatment strategies and outcomes for EVI over a nine-year period.

**Method: **A retrospective study of adult and paediatric patients admitted to a single UK Major Trauma Centre with vascular injuries to the upper and/or lower limbs between February 2012 and August 2021. Deidentified data was collected from the Trauma Audit and Research Network, and the hospital trauma registry and computer record system.

**Results: **EVI was identified in 399 patients (340 adults and 59 paediatric patients). The median age was 28 (21–40) years, 342 (85.7%) were male and 207 (51.9%) had a penetrating mechanism of injury. There were 250 (62.7%) patients with arterial injuries, 107 (26.8%) with arterial and venous injuries, whilst 42 (10.5%) only sustained venous injuries. First-line surgical techniques for all vessel types were primary repair (n = 108), ligation (n = 94), interposition grafts (n = 84), non-operative treatment (n = 72), embolectomy only (n = 19) and temporary intravascular shunts (n = 17). The median time to surgery was 2.9 (1.4–12.2) hours. The 30-day mortality rate was 7.3% (n = 29) and the in-hospital surgical amputation rate was 12.5% (n = 50), of which 15 were secondary amputations. Multivariate regression analysis revealed a blunt mechanism of injury (*p* < 0.0001) and intervention complications from vessel occlusion or bleeding post-repair (*p* = 0.007) were independent predictors of amputation. The amputation rate in recent years 2018–2021 was significantly lower compared to 2013–2017 (9.5% vs 16.3%; OR 0.539 (95% CI 0.292–0.995); *p* < 0.05).

**Conclusion: **Limb salvage and survival after EVI was achieved in the majority of the cohort. Potential predictors of amputation include blunt trauma and intervention complications. Increased limb salvage rates over time may reflect improvements in multidisciplinary input.

## P36 Pre-hospital traumatic cardiac arrest: an evaluation of timelines and causal injuries in patients treated by London's Air Ambulance

### Aditi Nijhawan^1,2,*^, Jared Wohlgemut^2,3^, Zane Perkins^1,2,3^

#### ^1^London’s Air Ambulance, UK. ^2^Barts Health NHS Trust, UK. ^3^Centre for Trauma Sciences, Blizard Institute, Queen Mary University of London, UK

##### *Correspondence: aditi.nijhawan1@nhs.net

***Scandinavian Journal of Trauma, Resuscitation and Emergency Medicine*** 2023, **31(1)**: P36

**Background: **Traumatic cardiac arrest (TCA) is associated with poor outcomes, with reported survival rates ranging from 0 to 7.5% [1]. There is little understanding of what specific injuries lead to TCA, as most patients in TCA die on scene, limiting definitive follow up. Furthermore, there is no substantive literature on time from injury to arrest, despite this being the window for maximum impact of critical care teams. This study aims to understand causative injury patterns of TCA, and compare time from injury to arrest between various injuries.

**Methods: **A retrospective database review was conducted of all patients London’s Air Ambulance (LAA) attended between 01/01/2019 and 31/12/2021. All patients in prehospital TCA were included, excluding hanging, conflagration, electrocution, and conditions unequivocally associated with death [2]. Time of injury was estimated using ‘999 call time’ as a surrogate, unless a more accurate time was documented. Time of arrest was estimated from police, ambulance service or LAA records. Definitive injuries were determined by post-mortem, surgical, radiological, and clinical findings.

**Results: **During the three-year study period, LAA treated 4689 injured patients. Of these, 628 (13.4%) had prehospital TCA and 514 (11.0%) met the study eligibility criteria. Median (IQR) time from injury to arrest in minutes was 12 (6–25) overall, 8 (5–30) for blunt and 13 (6–21) for penetrating trauma; p = 0.53. Median (IQR) time was 9.5 (6–15) for cardiac injuries and 15 (10.5–20.5) for major arterial injuries; p = 0.078. The commonest injuries causing TCA after blunt trauma were central nervous system injuries and after penetrating trauma were chest and neck injuries.

**Conclusion: **For patients who suffer pre-hospital TCA, the rapidity of deterioration is such that even the most efficient teams will rarely arrive on scene in time to prevent TCA. Pre-hospital clinicians must be prepared to rapidly assess and treat TCA, while understanding the baseline causes.

**References**

1. David Lockey, Kate Crewdson, and Gareth Davies. ‘Traumatic Cardiac Arrest: Who Are the Survivors?’ *Annals of Emergency Medicine* 48, no. 3 (1 September 2006): 240–44..
2. Joanne Fisher, Simon Brown, and Matthew Cooke. ‘UK Ambulance Service Clinical Practice Guidelines (2006)’. *Joint Royal Colleges Ambulance Liaison Committee (JRCALC)*, 2006, 428.

## P37 Emergency checklist use in single-pilot air ambulance operations: a safety analysis in cockpit among two comparative groups of crew members

### Håvard Mattingsdal^1^,*, Andreas Hjert^2^

#### ^1,*^Faculty of Science and Technology, University of Stavanger, Stavanger, Norway. ^2^Flight Operations Department, Norwegian Air Ambulance, Oslo, Norway

##### *Correspondence: haavard.mattingsdal@norskluftambulanse.no

***Scandinavian Journal of Trauma, Resuscitation and Emergency Medicine*** 2023, **31(1)**: P37

**Background: **Helicopter Emergency Medical Services (HEMS) are operated either as a single-pilot with HEMS technical Crew Member (HCM)- or a two-pilot system. Norwegian HEMS is a single-pilot operation with a HCM and a pilot in the cockpit, aiming for a level of competence and safety approximating a two-pilot system. This carries a continuous focus on safety and redundancy in the event of an acute illness, i.e. “pilot incapacitation” [1]. Currently, the service lacks emergency procedures for such an incident. The solution could be to introduce checklists adapted to the HCM for such emergency situations. We aimed to analyse how a newly developed checklist, where the HCM utilizes the helicopter's autopilot technology, affects safe handling and landing during a simulated pilot incapacitation.

**Method: **Experimental study in a flight simulator during Instrument Meteorological Conditions (IMC) comparing two groups of HCMs. One control group (n = 3) operated according to the current standard without a checklist, compared to an intervention group (n = 3) using the new checklist. The safety analysis included the Systematic Human Error Reduction and Prediction Approach (SHERPA), objective workload with Heart Rate Variability (HRV) and subjective workload with the NASA Stress Load Index (TLX).

**Results: **The study showed that the HCMs using the new checklist had significantly fewer recorded human errors (21 versus 80 incidents, p < 0.05), no evidence of differences in recorded objective workload (12.1 versus 14.2 in stress index, *p* = 0.48) and significant differences in subjectively reported workload (22.5 versus 57.5 in general workload, *p* < 0.05), compared to the group that operated according to today's standard.

**Conclusion: **The study shows that a standardized checklist contributes to increased safety during pilot incapacitation in a single-pilot with HCM operation. Introduction of this new checklist in Norwegian HEMS is realistic due to the existing competence of the HCMs and available technology in the cockpit.

**Reference**

1. Huster KM, Muller A, Prohn MJ, Nowak D, Herbig B. Medical risks in older pilots: a systematic review on incapacitation and age. Int Arch Occup Environ Health. 2014;87(6):567–578.

## P38 An ambulatory pathway for patients presenting with spontaneous pneumothorax at Oxford University Hospitals

### Eva Howard^*^

#### Emergency Medicine Specialist Registrar, Defence Deanery, UK

##### *Correspondence: evahoward21@gmail.com

***Scandinavian Journal of Trauma, Resuscitation and Emergency Medicine*** 2023, **31(1)**: P38

**Background: **The British Military is one of the UK’s largest employers of divers, air crew and parachutists, all of whom have specific job-dependent restrictions regarding pneumothorax. They are also the demographic most at risk of spontaneous pneumothorax; often young males who smoke. This quality improvement project developed an ambulatory pathway for patients presenting to Oxford University Hospitals (OUH) with primary spontaneous pneumothorax (PSP).

**Methods: **Analysis / Understanding of the issue: The ambulatory pathway was developed based on latest evidence from the Lancet paper by Hallifax et al. [1], combined with expert opinion including Respiratory, Medical and Emergency Medicine consultants at OUH. Prior to the ambulatory pathway, it was identified that there were 79 patients presenting to OUH with PSP, who combined spent 174 nights in hospital. The majority of PSP patients are otherwise mostly fit & clinically well, therefore it was identified that some of these nights spent in hospital were unnecessary & the RAMP trial had provided evidence that most of these patients could have been treated at home safely. Change implementation: The pathway underwent approval by the departmental clinical governance committee and a number of products were created to provide visual schematics aimed at various clinicians. Teaching sessions were also delivered, increasing awareness of the new pathway including use of the Rocket Pleural Device so patients could be discharged even whilst their pneumothorax was still draining; a mindset shift. QI Methodology: The primary outcome measure was reduction in length of in-hospital stay. The balance outcome measures were patient satisfaction and adverse events. These were measured by data from electronic health records and a patient satisfaction questionnaire.

**Results: **163 patients were identified as being treated for spontaneous pneumothorax at OUH during the two year study period (one year prior to and one year after the instigation of the ambulatory pathway). Initial data shows a statistically significant reduction in nights spent in hospital with 78 of the 83 patients with PSP being ambulated for at least part of their treatment, in total spending only 43 nights in hospital. This was a reduction of 131 nights. There was also an increase in patient satisfaction and no increase in adverse events.

**Conclusions: **This has now become an established pathway within OUH with over 85% of PSP patients ambulated last year. Ambulatory pneumothorax training for 2022 has already commenced and the schematic has been shared with other Emergency Departments around the country.

**Reference**

1. Hallifax RJ, et al. Ambulatory management of primary spontaneous pneumothorax: an open-label randomised control trial. Lancet. 2020 Jul 4;396(10,243):39

## P39 Prehospital tracheal intubation: assessing the risk of endotracheal tube migration during transfer.

### Victoria Lee^1,*^, Amar S. Jessel^2^, Laura May^3^

#### ^1,*^Anaesthetics & PHEM Speciality Registrar, University Hospital Coventry & Warwickshire. ^2^Anaesthetics Speciality Registrar, Birmingham Children’s Hospital. ^3^University Hospital Coventry & Warwickshire

##### *Correspondence: doctorvictorialee@gmail.com

***Scandinavian Journal of Trauma, Resuscitation and Emergency Medicine*** 2023, **31(1)**: P39

**Background: **Tracheal tube (TT) misplacement is a recognised and common complication of intubation, including inadvertent endobronchial intubation & oesophageal intubation.^1^ We reviewed the documented insertion depth of TTs from prehospital intubations in adult patients and compared these to documented TT depth upon arrival to our Major Trauma Centre in order to assess the incidence of migration during transfer.

**Method: **Data was collected over approximately a 2.5 year period for all patients following a prehospital intubation and transfer to University Hospitals Coventry & Warwickshire. All adults over the age of 16 years were included, who were primary transfers and were either trauma or non-trauma patients. Goodman’s criteria was used for standard comparison of TT insertion depth; whereby the distance of the TT from the carina should be 5 cm ± 2, with TT reference mark of 23 cm for men & 21 cm for women.^2^ Patient notes were examined and recorded TT depth at discrete entries for different episodes in the patient’s pathway.

**Results: **117 patients underwent a prehospital emergency anaesthetic (PHEA) during this time period with only 32 of these patients having complete data sets. Of these, 22 had different TT depths noted during their journey, giving a potential incidence of TT migration of 67%. Ten of these were documented to have gone on to be purposefully manipulated.

**Discussion: **This audit has revealed that within our data set, we have evidence of TT misplacement, a proportion of which go unrecognised. The clinical implication of this is unclear, but unintentional endobronchial intubation can lead to complications such as hypoxia and pneumothoraces.^3^ Our audit has raised a key consideration; what can we do to reduce the incidence of TT misplacement, or increase early detection, to maintain patient safety?

**References**

1. Ono Y, Kakamu T, Kikuchi H, Mori Y, Watanabe Y & Shinohara K. Expert-performed endotracheal intubation-related complications in trauma patients: Incidence, possible risk factors, and outcomes in the prehospital setting and emergency department. *Em Med International*, 2018. https://doi.org/10.1155/2018/5649476
2. Ong KC, A’Court GD, Eng P, Ong PP. Ideal endotracheal tube placement by referencing measurements on the tube. *Annals Academy Medicine*, 1996, 25(4):550–2.
3. Al-Qahtani A, Messahel F, Ouda W. Inadvertant endobronchial intubation: A sentinel event. *Saudi J Anaesth.* 2012, 6(3):259–62

## P40 Improving performance in helicopter emergency services (HEMS) and helicopter search and rescue (HSAR); Experiences from the Royal Norwegian Air Force 330 Sqn

### Sven C Skaiaa^1,*^, Mads Sabel^1^, Dag S Jakobsen^2^, Lars Markengbakken^2^, Jens Klüver^2^, Per-Olav Berve^1^

#### ^1,*^Air Ambulance Dept., Oslo University Hospital, Oslo, Norway. ^2^330 Sqn SAR helicopter services, Sola, Norway

##### *Correspondence: scskaiaa@gmail.com

***Scandinavian Journal of Trauma, Resuscitation and Emergency Medicine*** 2023, **31(1)**: P40

**Background: **Rapid access, optimal timing of meaningful interventions, and efficient evacuation to definitive care is considered the mainstay in prehospital care. Attaining the team cooperation needed to efficiently link prehospital phases and their associated procedures into an overall Medical-Operative Workflow (MOW) remains a challenge during complex HEMS/HSAR missions. The performance of Critical Care and Rescue Teams (CART, a physician and a rescue-specialist) varies due to individual preferences and priorities. We hypothesized that introducing a common MOW tailored to our mission profile would improve mission safety, quality of care and transport logistics.

**Materials and methods: **From 2019 we aimed to describe, evaluate, and develop the MOW with respect to the 330 Sqn´s mission scope and Standard Operating Procedures. All phases from dispatch to patient delivery were analysed, including direct CART hoist insertion and extrication techniques. Cumulative experience, literature and individual work patterns were reviewed. Equipment set-up, physical organization, communication, and human factors were gradually evaluated, revised, and simulated to develop the most efficient workflows. The final suggested MOW was thereafter validated in full-scale scenarios utilizing video analysis to establish proof-of-concept.

**Results: **The “Medical Operative Workflow Manual (MOWM)”, that details the revised standardized work methodology for HEMS/HSAR, was published within the 330 Sqn in March 2022. Pilot courses and simulations for experienced CART´s (n = 9) were performed. All CART members reported improved cognitive capacity, safety focus and perceived ability to provide high quality care. Data from simulations, and later actual missions, indicate reduced dispatch, on-scene, and evacuation times.

**Conclusions: **Implementation of the MOWM has shown promising results on indicators of overall mission efficacy. This has led to an increased acknowledgement of the necessity for both describing and training detailed team workflow to further develop a high-performance HEMS/HSAR culture within our organization. Future research on HEMS/HSAR workflow development and implementation is needed.

## P41 Fast, but less furious; standardization of Medical Operative Workflow (MOW) to improve critical care in the Royal Norwegian Air Force 330 sqn Helicopter Emergency & Search and Rescue service (HEMS/HSAR)

### Per Olav Berve^*^, Mads Sabel, Sven Christjar Skaiaa

#### Air Ambulance Dept., Oslo University Hospital, Oslo, Norway

##### *Correspondence: poberve@gmail.com

***Scandinavian Journal of Trauma, Resuscitation and Emergency Medicine*** 2023, **31(1)**: P41

**Background: **Efficient and timely application of meaningful life-saving interventions are essential skills in pre-hospital care. Modern possibilities for diagnosis and treatment, including a plethora of published standard operating procedures, may unintentionally disrupt overall workflow. To maintain quality of care, speed and safety throughout complex missions, our recently developed Medical Operative Workflow Manual (MOWM) includes a standardized and easily trainable medical workflow for core prehospital procedures. In this proof-of-concept study we hypothesized that medical workflow training would reduce procedure times and increase treatment quality.

**Materials and methods: **Baseline procedure-times were identified through video analysis. Procedures and equipment set-up were revised over a two-year period including literature search, SIM-sessions, and drill. The suggested new workflows, time-goals and quality indicators were thereafter validated utilizing video-feedback. A right-sided setup was found to be most efficient: the main equipment bag above patient’s shoulder, the monitor-defibrillator beside patient’s thigh and the rescue-specialist placed between these. Data were captured from the video analysis of experienced, but MOWM-naïve, physician and rescue-specialist teams who were trained and tested in two simulated procedures: (1) Two-provider initialisation of ACLS: From patient arrival to secured airway (CPR, DC-shock, BVM-ventilation, capnometry, endotracheal tube placed, secured and tested). (2) Two-provider RSI: From patient arrival to hoist ready (patient moved into hoist-stretcher, hypothermia protection, full monitoring, clinical examination, IV-cannulation, anaesthesia, intubation, on mechanical ventilator).

**Results: **All teams (n = 9) performed both procedures correctly and within the prespecified time-goals: (1) Two-provider ACLS: < 1.5 min (baseline > 6 min), (2) Two-provider RSI-to-hoist ready: < 11 min (baseline > 20 min). All participants reported perceived increased performance and improved safety awareness.

**Conclusions: **Application of a standardized MOW enabled a substantial time-improvement in performing two core medical HEMS-procedures, with maintained procedural quality. The results suggest that implementation of MOW in HEMS/HSAR should be both emphasized and studied in the future.

## P42 The role of CT in traumatic aortic injury in a large teaching hospital

### Khalaf A. Alshamrani^1,*^, Fawaz F. Alqahtani^1^

#### ^1^Department of Radiological Sciences, Najran University, Saudi Arabia

##### *Correspondence: Kaalshamrani@nu.edu.sa

***Scandinavian Journal of Trauma, Resuscitation and Emergency Medicine*** 2023, **31(1)**: P42

**Background: **Traumatic aortic injury (TAI) is considered as an emergency and a life-threatening condition that requires prompt diagnosis and management. Computed tomography (CT) is the current method of choice for assessing traumatic injuries. However, early studies of CT scans reported limited sensitivity for the detection of TAI.

**Objectives: **To study the role of CT scan in diagnosis of traumatic aortic injury and to determine the commonest findings seen in CT scan in patients with thoracic injury, head and abdomen injury.

**Materials: **A retrospective cross-sectional study was conducted in a large teaching hospital at Eastern region in Saudi Arabia. All patients with traumatic aortic injury within 2-years, who had undergone CT scan were included in the study.

**Results: **Total of 234 medical record were reviewed in which 65 patients were included in this study. From the 35 patients with aortic injuries, (94.3%) were males. The commonest age group was found to be 20–35 years (48.6%), followed by 36–50 years. Road Traffic Accident was the commonest cause of injury (77.1%), followed by blast shot (11.5%). Regarding injury’s grade, the commonest grade was found to be grade II (40%), followed by grade IV (31%). Head injuries were seen in 60% of the injuries while the abdominal aorta is involved in only approximately 15% of TAI cases.

**Conclusion: **Traumatic injury of the thoracic aorta remains the leading cause of death in multiple trauma patients and it requires urgent management. CT scanners with its high accuracy and high sensitivity can clearly display the aortic and other associated injuries. Within this study, it appears that RTA are the most cause of aortic injury.

## P43 Rationale and methods of the Antioxidant and NMDA receptor blocker Weans Anoxic brain damage of KorEa OHCA patients (AWAKE) trial

### Jin-Ho Choi^1,*^, Byeong Jo Chun^2^, Seok Ran Yeom^3^, Sung Phil Chung^4^, Young Hwan Lee^5^, Yun-Hee Kim^6^, Jin Sung Lee^7^, Jin Hwan Lee^8^, Hwan Goo Lee^8^, Jing Yu Jin^8^, Chun San An^8^, Byoung Joo Gwag.^8^

#### ^1,*^Samsung Medical Center, Department of Emergency Medicine, Sungkyunkwan University School of Medicine, Seoul, Republic of Korea. ^2^Chonnam National University Hospital, Department of Emergency Medicine, Chonnam National University Medical School, Gwangju, Republic of Korea. ^3^Pusan National University Hospital, Department of Emergency Medicine, Pusan National University School of Medicine, Busan, Republic of Korea. ^4^Gangnam Severance Hospital, Department of Emergency Medicine, Yonsei University College of Medicine, Seoul, Republic of Korea. ^5^Department of Emergency Medicine, Soonchunhyang University Bucheon Hospital, Bucheon‑si, Gyeonggi‑do, Republic of Korea. ^6^Samsung Medical Center, Department of Physical and Rehabilitation Medicine, Sungkyunkwan University School of Medicine, Seoul, Republic of Korea. ^7^Department of Clinical Epidemiology and Biostatistics, Asan Medical Center, University of Ulsan College of Medicine, Seoul, Korea. ^8^GNT Pharma Co. Ltd, Yongin‑si, Gyeonggi‑Do, Republic of Korea

##### *Correspondence: jhchoimd@gmail.com

***Scandinavian Journal of Trauma, Resuscitation and Emergency Medicine*** 2023, **31(1)**: P43

**Background: **Ischemic brain injury is a major hurdle that limits the survival of resuscitated out-of-hospital cardiac arrest (OHCA).

**Methods: **The aim of this study is to assess the feasibility and potential for reduction of ischemic brain injury in adult OHCA patients treated with high- or low-dose Neu2000K, a selective blocker of N-methyl-d-aspartate (NMDA) type 2B receptor and also a free radical scavenger, or given placebo. This study is a phase II, multicenter, randomized, doubleblinded, prospective, intention-to-treat, placebo-controlled, three-armed, safety and efficacy clinical trial. This trial is a sponsor-initiated trial supported by GNT Pharma. Successfully resuscitated OHCA patients aged 19 to 80 years would be included. The primary outcome is blood neuron-specific enolase (NSE) level on the 3rd day. The secondary outcomes are safety, efficacy defined by study drug administration within 4 h in > 90% of participants, daily NSE up to 5th day, blood S100beta, brain MRI apparent diffusion coefficient imaging, cerebral performance category (CPC), and Modified Rankin Scale (mRS) at 5th, 14th, and 90th days. Assuming NSE of 42 ± 80 and 80 ± 80 μg/L in the treatment (high- and low-dose Neu2000K) and control arms with 80% power, a type 1 error rate of 5%, and a 28% of withdrawal prior to the endpoint, the required sample size is 150 patients.

**Discussion: **The AWAKE trial explores a new multi-target neuroprotectant for the treatment of resuscitated OHCA patients.

*Trial registration*: ClinicalTrials.gov NCT03 651557. Registered on August 29, 2018.

## P44 A five-year analysis of traumatic deliberate self-harm admissions to a Major Trauma Centre: does sex matter?

### Laura Zagarella, Max Marsden, Elaine Cole

#### Centre For Trauma Sciences, Barts and the London School of Medicine & Dentisty, Queen Mary University of London, London, United Kingdom

***Scandinavian Journal of Trauma, Resuscitation and Emergency Medicine*** 2023, **31(1)**: P44

**Background: **Deliberate self-harm (DSH) and suicide are common and preventable. There is limited data on the epidemiology of DSH, including between sexes. The aims of this study were to explore the sex differences in DSH admission frequency, mechanism of injury, demographics, outcomes and explore temporal changes.

**Methods: **A retrospective analysis of local trauma registry data was conducted for all DSH presentations that triggered a trauma team activation at the Royal London Hospital (RLH) from 2015 to 2020. Statistical comparisons were performed to assess for differences between DSH patients based on sex and over time.

**Results: **In total 18,407 trauma patients attended RLH between 2015 and 2020, of which 968 patients (5.3%) were injured as a result of DSH. There was no significant variation in the proportion of DSH patients’ year-on-year. Overall, two thirds (592, 61%) of the patients were male and the proportion of males presenting with DSH varied each year (51–76%). Within sex groups, the proportion of trauma that resulted from DSH in female patients was almost twice that of males (9% vs. 4%, *p* < 0.01). Penetrating injury was the most common mechanism of injury in both sexes and more prevalent in females (59% vs. 51%, *p* = 0.009). After inpatient admission, men had a greater probability of critical care admission (F: 17% vs. M: 28%, *p* = 0.0002) whilst females were more likely to be discharged home (F: 62% vs. M: 47%, *p* = 0.0001). Males had a three-fold increase in complete suicide (2% vs 6% *p* = 0.002).

**Conclusions: **Approximately 1 in 20 trauma call activations at the RLH were a result of a DSH injury. More men were injured due to DSH and these men had worse outcomes. However, between sex groups a greater proportion of women were injured because of DSH than men. This study provides useful information to help target preventative strategies to reduce DSH and suicide.

## P45 Comparing outcomes for early vs late formal surgical debridement of bites to hands: a retrospective cohort study

### Mohamed Eldolify*, Belal Darwiche, Ripak Purbe

#### *Queen’s Medical Centre, Nottingham University Hospitals NHS Trust, United Kingdom

##### *Correspondence: Mohamed.eldolify@nhs.net

***Scandinavian Journal of Trauma, Resuscitation and Emergency Medicine*** 2023, **31(1)**: P45

**Background: **Bite injuries are extremely common presentations, and the most commonly encountered culprits are dogs (80%), followed by cats and humans [1]. Bites to hands are particularly more prone to infection, often requiring formal surgical debridement [2]. Current evidence is lacking for the timing of said debridement, and our aim with this study was to compare outcomes associated with early (< 24 h from presentation; group I) and late (> 24 h from presentation; group II) surgery.

**Methods: **This is a retrospective cohort study examining patient records in a busy teaching hospital in Nottingham, UK. A total of 412 cases were identified as having presented with bites (Dog, cat, or human etiology only) to the hand, out of which 22 patients met the criterion of having formal operative surgical debridement. Most variables including delay in presentation, co-morbidities, site and severity of injury and the initial management were considered and accounted for in both groups, and specified short- and long-term outcomes were identified and measured.

**Results: **Results showed statistically significant difference for need for second procedure (none of group I vs 33% of group II, *p*-value = 0.0245), prolonged hospital stay following the surgery (*p* = 0.0245) and deep infection (22% vs none in group I, *p* = 0.0810). Neither group developed osteomyelitis, osteoarthritis, or long-term complications.

**Discussion: **The small sample size caused limitations, and generalization cannot be reliably made. However, current practice is based on anecdotal findings, and there is not enough evidence currently available. We hope that this study will provide more evidence to guide future recommendations.

**Reference**

1. Kennedy, S.A., Stoll, L.E. and Lauder, A.S. (2015) Human and other mammalian bite injuries of the hand: evaluation and management. Journal of the American Academy of Orthopaedic Surgeons 23(1), 47–57.2. National Institute for Clinic Excellence (NICE) Clinical Knowledge Summaries: Bites- Human and Animal, last revised August 2021 [accessed June 2022]. Available from: https://cks.nice.org.uk/topics/bites-human-animal/

## P46 An enhanced algorithm for refractory shockable rhythms terminates VF/VT and improved outcomes in out of hospital cardiac arrest

### James Raitt^*^, Chloe Spence, Nick Cole, Mark Hodkinson, Jo Jefferies, Kurt Poole, Jon Bailey, Asher Lewinsohn

#### ^*^Thames Valley Air Ambulance, Stokenchurch, UK

##### *Correspondence: James.Raitt@tvairambulance.org.uk

***Scandinavian Journal of Trauma, Resuscitation and Emergency Medicine*** 2023, **31(1)**: P46

**Background: **Management of cardiac arrest according to published guidelines has remained largely unchanged for a decade. Thames Valley Air Ambulance provide Critical Care Paramedic and Physician teams who respond to cardiac arrests and offer treatments beyond the scope of ambulance service clinicians. Following a review of practice and appraisal of evidence we developed an additional algorithm for cases of adult medical cardiac arrest with refractory shockable rhythms which adds to but does not replace the Advanced Life Support algorithm and includes:Delivering shocks with LUCAS mechanical CPR running. After 5 shocks placing pads Anterior Posterior (AP).

**Method: **This was an observational study, cases were analysed for the 6 months following the introduction of the algorithm (October 2021–March 2022) and compared to the same period the year before.

**Results: **In the 6 months prior to implementation there were 9 cases of refractory shockable rhythms and the median number of shocks was 13. In the 6 months following the introduction of the refractory shockable rhythm algorithm 13 cases met the inclusion criteria, a median of 7 shocks were given prior to AP pads being applied. In 6 out of 13 cases the refractory rhythm was terminated with 1 AP shock, in 3 cases 2 shocks were required and in four cases 3 or more AP shocks were required. The median number of AP shocks was 2. The rate of ROSC at hospital handover for the intervention group was 31%, compared to 11% for the period of standard care only.

**Conclusion: **The enhanced algorithm has increased the rate of ROSC at hospital handover and resulted in termination of the majority of refractory shockable rhythms with 2 or fewer AP shocks. This study adds significantly to the limited existing evidence, wider dissemination of the techniques and a larger study are warranted.

## P47 A multimodal approach to lateral canthotomy and cantholysis training for Emergency Medicine trainees

### Yunus K Hussain^1,*^, Cara Jennings^2^, Tara Smith.^2^

#### ^1,*^Department of Paediatrics, Basildon and Thurrock University Hospital, Basildon, Essex, UK. ^2^Emergency Department, King’s College Hospital, London, UK

##### *Correspondence: yunus.hussain@outlook.com

***Scandinavian Journal of Trauma, Resuscitation and Emergency Medicine*** 2023, **31(1)**: P47

**Background: **Lateral canthotomy and cantholysis (LCC) is a sight-saving procedure for orbital compartment syndrome (OCS). Emergency medicine trainees must be able to competently conduct this procedure. In a questionnaire we found that trainees have low confidence levels in performing the procedure attributed to the low incidence of OCS and the scarcity of training opportunities. Currently, no standardised economical simulators or practical education packages are available for procedural training. We designed and tested a multimodal training approach to optimise trainee confidence and competence in recognising OSC and performing the LCC procedure.

**Method: **The training sessions were delivered as part of a regional procedural skills course for ST3 Emergency Medicine trainees. The sessions consisted of a PowerPoint presentation outlining the aetiology of OCS and the indications, methodology, complications and aftercare of LCC. We used a modified form of the low-fidelity simulator designed by Kong et al. [1], and created a video demonstrating the procedure using this model. Trainees then had the opportunity to practice the procedure on the models. To consolidate and assess the trainees' knowledge, we designed and delivered a short MCQ using Kahoot!.™ Trainees’ confidence and perceived competence in conducting the procedure before and after the training were assessed using a 10-point Likert scale.

**Results: **Fifteen participants attended the sessions 80% of whom had either no or only e-learning exposure to the LCC procedure. We demonstrated an 80% and 86.7% improvement in participants achieving high confidence levels in their theoretical knowledge and procedural competence respectively. All participants reported that this training package should be included as part of annual ST3 trainee training.

**Conclusions: **Given the severity of OCS, there is a need for diagnostic and procedural competence which we have shown to be enhanced following completion of this training package. We aim to deliver this training package annually for ST3 training.

**Reference**

1. Kong R, Kaya DP, Cioe-Pena E, Greenstein J. A low fidelity eye model for lateral canthotomy training. *Afr J Emerg Med*. 2018; 8(3):118–122. https://doi.org/10.1016/j.afjem.2018.02.002. PMID: 30,456,160.

## P48 Trends in Trauma Mortality and the Human Factors influencing Preventable Deaths: A Mixed-method, Single-centre Retrospective Study

### R. Bradley^1,*^, J. Wolghemut^1^, A Lappin^2^, A Rose^1^, M Edwards^1^

#### ^1,*^Royal London Hospital, Barts Health Trust, London, UK. ^2^Department of Computer Sciences, University of Warwick, Coventry, UK

##### *Correspondence: Rebecca.bradley16@nhs.net

***Scandinavian Journal of Trauma, Resuscitation and Emergency Medicine*** 2023, **31(1)**: P48

**Background: **Mortality after trauma has reduced over time. A review of mortality in Europe’s busiest Major Trauma Centre (MTC) is due. The concept of ‘preventability’ is a standard method of measuring the quality of trauma care. ‘Preventable’ deaths require scrutiny to improve trauma provision and identify aspects of care which are vulnerable to human error.

**Methods: **A mixed-methodology single-centre retrospective cohort study was conducted. Consecutive trauma patients who were admitted to a London MTC and died between 01.01.2016 and 31.12.2021 were included. Demographics, injury patterns and Ps were described. Deaths were classified: non-preventable (NPD), potentially preventable (PPD) or preventable (PrevD). A subgroup of 24 random patients was selected for human factors framework evaluation, to identify ‘deviations from the expected care pathway’ (DECPs).

**Results: **Of the 776 deaths, 668 met study inclusion criteria. Median age was 65.7 (IQR 38.5–82.8). More males than females died. The most common mechanism of injury was ‘fall < 2 m’ (n = 256). There were 283 (42.4%) NPDs, 143 (21.4%) PPDs, and 242 (36.2%) PrevDs. Characteristics in PrevDs which were significantly included: increasing age (*p* = 0.03), transferred patients, (p =  < 0.001), decreasing ISS (*p* ≤ 0.001) and fall < 2 m (< 0.001). Human factors framework analysis showed patients were most likely to have a DECP on the ward than at any other point in their journey. ED was the second most vulnerable area. Common DECPs identified included delayed specialty review, disregarding guidelines, delayed imaging reports, and lack of senior review.

**Conclusions: **Approximately 1/3 of all MTC deaths were preventable. Preventable deaths were most prevalent in older patients, falls < 2 m, and with a lower injury severity. From our results, there is a need for further investigation for patients on the ward and those in ED. Further qualitative work is required to identify the reasons for each DECP found. Improvements in these areas could reduce the number of preventable deaths.

## P49 Emergency medical communication center assessment of suspected and verified stroke patients

### Bjørn Jamtli^1,2,*^, Maren R Hov^1,2^, Trine M Jørgensen^2^, Jo Kramer-Johansen^1,3^, Hege Ihle-Hansen^1^, Else C Sandset^1,4^, Camilla Hardeland^1,5^

#### ^1^Oslo University Hospital, Oslo, Norway. ^2^Oslo Metropolitan University, Oslo, Norway. ^3^University of Oslo, Oslo, Norway. ^4^Norwegian Air Ambulance Foundation, Oslo, Norway. ^5^Østfold University College, Fredrikstad, Norway

##### *Correspondence: Bjorn.Jamtli@helsedir.no

***Scandinavian Journal of Trauma, Resuscitation and Emergency Medicine*** 2023, **31(1)**: P49

**Background: **Stroke recognition in Emergency Medical Communication Center (EMCC) reduces prehospital delay and improve outcome (1). Several studies have documented substantial variations in EMCC stroke recognition rates, but few studies have identified specific measures for improvement. In this study, we aimed to explore EMCC assessment of emergency calls from suspected and verified stroke patients and to identify the potential for improvement of EMCC stroke recognition.

**Method: **We performed a descriptive retrospective study, based on emergency department and EMCC records from a comprehensive stroke center in Oslo, Norway. Patients dispatched with EMCC stroke criteria and/or discharged with a stroke diagnosis after initial EMCC assessments were included.

**Results: **During the six-month study period (2019–2020) we identified 1298 patients eligible for analysis, of which 20% were discharged with a main diagnosis of stroke. 77% of all stroke patients were dispatched with stroke criteria, but only 16% of all patients dispatched with stroke criteria were diagnosed as stroke at hospital discharge. Stroke patients not identified by EMCC had a 10 min additional prehospital delay. Only 2.2% of all patients dispatched with a stroke criteria and referred to GP, out of hours GP or left on scene by the ambulance services were diagnosed as stroke patients. As a consequence, we argue that both EMCC false negative and EMCC false positive stroke patients not referred to hospital by the ambulance service represents the main potential for improvement on EMCC stroke sensitivity and specificity.

**Conclusion: **This study reports high EMCC stroke sensitivity, an extensive number of false positive stroke dispatches and a moderate number of false negative stroke dispatches. EMCC false negative and false positive stroke patients not referred to hospital by the ambulance service represents the main potential for improvement on EMCC stroke recognition and should be focus for further research.

**Reference**

1. J. E. Acker, 3rd, A. M. Pancioli, T. J. Crocco, M. K. Eckstein, E. C. Jauch, H. Larrabee, et al.Implementation strategies for emergency medical services within stroke systems of care: a policy statement from the American Heart Association/ American Stroke Association Expert Panel on Emergency Medical Services Systems and the Stroke Council. Stroke 2007 Vol. 38 Issue 11 Pages 3097–115 https://www.ncbi.nlm.nih.gov/pubmed/17901393

## P50 The “Code Black” experience at King’s College Hospital Emergency department

### Siddharth Sinha^1,*^, Alexander Robertson^1^, Katie McLeod^1^ and Malcolm Tunnicliff^1^

#### ^1,*^Emergency Department, King’s College Hospital, London, Greater London, United Kingdom

##### *Correspondence: Siddharth.sinha@nhs.net

***Scandinavian Journal of Trauma, Resuscitation and Emergency Medicine*** 2023, **31(1)**: P50

**Background: **Code black is a criteria used to identify potential neurosurgical emergencies in the pre-hospital setting or upon arrival to the emergency department. If activated patients are taken directly to computerised tomography (CT) scanner for rapid assessment and imaging, bypassing Resus. Code black is triggered if there is evidence of raised intracranial pressure, clinical suspicion of head injury, a Glasgow coma scale of less than 8 and code red is not triggered. Here we present our findings of the criteria at King’s College London.

**Methods: **We retrospectively analysed patients who activated the code black protocol 6 months after the establishment of the criteria in August 2021. We compared the “code black” group to a matched cohort of isolated head injury patients 6 months prior to the criteria being put in place. Parameter assessed included; time from 999 call to CT scanner and the operating room. We also assessed the time from arrival to the CT scanner and the operating room.

**Results: **Our dataset included 16 “code black” patients and 11 “pre-code black” patients. We found that the code black protocol significantly reduced arrival to CT scanner (15 versus 22.2 min, *p* ≤ *0.001*). A near-significant reduction in time from arrival to the operating room was also evident (91.8 versus 214.2 min, *p* = *0.056*). No difference was found between the time of 999 call to arrival, CT scanner or to the operating room.

**Conclusion: **The “Code black” protocol shows early promise in reducing the time from arrival to CT scanner and to the theatre. Due to pre-hospital factors out of our control such as treatment time on-scene and transfer distances, reduction time from 999 call to arrival, imaging and theatre are less significant. This protocol can greatly impact how traumatic neurosurgical emergencies are managed in the emergency department.

## P51 Epidemiological Evaluation and Causes of delayed presentation of Orthopaedic polytrauma patients to Emergency Department—A Tertiary Care Centre Experience.

### Shiv S Tripathi^1,*^, Swagat Mahapatra^2^

#### ^1,*^Department of Emergency Medicine, Dr RMLIMS, Lucknow, Uttar Pradesh, India. ^2^Department of Orthopaedics, Dr RMLIMS, Lucknow, Uttar Pradesh, India

##### *Correspondence: shiv_shanker2@rediffmail.com

***Scandinavian Journal of Trauma, Resuscitation and Emergency Medicine*** 2023, **31(1)**: P51

Presented but previously published work.

## P52 Neuron-Specific Enolase: Standardised Sampling Method to a “No Needle” Technique to Combat High Rates of Haemolysis

### Oliver Crowther, Sarah Hillman, Jerry P. Nolan

***Scandinavian Journal of Trauma, Resuscitation and Emergency Medicine*** 2023, **31(1)**: P52

Royal United Hospitals Bath, UK

**Background: **Neuron-specific enolase (NSE) is a biomarker of neuronal ischaemia and forms part of a multimodal approach to neuro-prognostication following cardiac arrest [1]. High absolute (> 60 ug L^−1^) or increasing NSE values on serial measurements predict poor neurological outcome [1,2]. Unfortunately, NSE is not specific to neurones and is also found in red blood cells, for this reason, haemolysis can lead to sample rejection [1]. In our intensive care unit (ICU) we noted a rise in sample rejection due to haemolysis, which we aimed to address.

**Methods: **A retrospective study of ICU cardiac arrest patients between January 2017 and December 2021. Cases were identified from ICNARC coding and then cross-referenced against NSE samples. Patient demographics, and numbers of accepted and rejected NSE samples. A “no-needle” sampling technique was proposed; gentle aspiration of blood from a central venous catheter and injecting blood directly into the bottle. We delivered an educational campaign involving posters and “tea trolley” teaching. Data were collected using feedback questionnaires. Identical methodology was used for post-intervention analysis between January-May 2022.

**Results: **174/223 cases were included, 62% were out of hospital cardiac arrests (OHCA) and 40% had an initial shockable rhythm. NSEs were sent in 121 cases (OHCA 83% vs. in hospital cardiac arrest (IHCA) 47%). The rejection rate of 365 samples was 35%. There were 56 participants in the educational intervention with a feedback return rate of 93%. Mean Likert scores increased from 3.3 to 4.7/5 (“what is an NSE?”) and 2.7 to 4.1/5 (“how to take an NSE”). 10 cases were identified post-intervention, 90% had NSEs sent with a rejection rate of 19%.

**Discussion: **We identified a significant issue of NSE sample rejection due to haemolysis. With a “no-needle” technique, combined with education, we achieved an immediate improvement in staff confidence which translated into a reduction in haemolysis rates.

**References**

1. Nolan JP, Sandroni C, Bottinger, BW et al. European Resuscitation Council and European Society of Intensive Care Medicine guideline 2021: post-resuscitation care, *Intensive Care Medicine,* 47:369–421.
2. Sandroni C, D’Arrigo S, Cacciola S et al. Prediction of poor neurological outcome in comatose survivors of cardiac arrest: a systematic review, *Intensive Care Medicine,* 46:1803;1851

## P53 Validation of the Adapted Clavien Dindo in Trauma (ACDiT) scale to grade management related complications at a level I trauma center

### Niladri Banerjee^1,*^

#### ^1,*^Department of General Surgery, All India Institute of Medical Sciences, Jodhpur, India

##### *Correspondence: docniladri@gmail.com

***Scandinavian Journal of Trauma, Resuscitation and Emergency Medicine*** 2023, **31(1)**: P53

Presented but previously published work.

## P54 North West Air Ambulance (NWAA): Service evaluation following the introduction of a night car

### Sophie Riley^1,*^, Scott Beattie^2^, Andy Curran^3^

#### ^1^Medical student, University of Manchester, Manchester, UK. ^2^Pre-Hospital Emergency Medicine Consultant, North West Air Ambulance, Manchester, UK. ^3^Medical Director, North West Air Ambulance, Manchester, UK

##### *Correspondence: sophie.riley@doctors.org.uk

***Scandinavian Journal of Trauma, Resuscitation and Emergency Medicine*** 2023, **31(1)**: P54

**Background: **Pre-hospital Emergency Medicine describes the delivery of immediate medical care before a patient reaches the hospital setting [1]. The North West Air Ambulance (NWAA) charity is a service which provides enhanced pre-hospital care to critically ill and injured patients across the North West region of England. [2] In October 2021, the NWAA service introduced a night car, which is operational on a Friday and Saturday night from 1800 to 0200.

**Method: **This service evaluation collected data from the initial 6-month period during which the night car operated as part of the NWAA service. The data, which looked at activations, staffing, patient demographics, mission types and locations, interventions and drugs, was retrieved retrospectively from the service database, HEMSbase.

**Results: **The NWAA was able to mobilise a car for 48 out of 52 shifts, providing 92% coverage. Over the 6-month period, 81% of shifts were staffed by both a doctor and paramedic. In total, the night car had 190 activations, from which the team attended 58 patients. The most common mission type was assault (32.6%), closely followed by road traffic collision (32.1%). Over the course of the study, the night car team carried out 10 rapid sequence inductions (RSIs), 9 thoracostomies, 3 thoracotomies and administered blood products to 8 patients.

**Conclusion: **Results from this study suggest that the introduction of a night car has had a beneficial impact on the service. Within the North West region, it has increased the provision of enhanced prehospital care, allowing a significant number of interventions to be carried out. This has included rapid sequence inductions (RSIs), delivery of blood products, thoracotomies and thoracostomies. On this basis, it is recommended that the night car continues to operate and further investment is made to ensure it is a reliable and robust service, aiming for 100% coverage 7 days a week.

**Acknowledgements**

North West Air Ambulance (NWAA) crew and trustees. This project was approved as a Service Evaluation by the North West Ambulance Service (NWAS).

**References**

1. General Medical Council (GMC). *Pre-hospital emergency medicine curriculum*. 3^rd^ edition. Available at: https://www.gmc-uk.org/education/standards-guidance-and-curricula/curricula/pre-hospital-emergency-medicine-curriculum [Accessed: 31st August 2022]
2. North West Air Ambulance Charity. *Our Story. Flying to save lives.* https://www.nwairambulance.org.uk/what- we-do/about-us/our-story/ [Accessed: 31st August 2022].

## P55 Dispatch of critical care assets to out-of-hospital cardiac arrest: DOHCA15 & DOHCA30

### Mark Hodkinson*, Kurtis Poole

#### Thames Valley Air Ambulance, Stokenchurch House, Oxford Road, Stokenchurch. HP14 3SX. UK

##### *Correspondence: mark.hodkinson@tvairambulance.org.uk

***Scandinavian Journal of Trauma, Resuscitation and Emergency Medicine*** 2023, **31(1)**: P55

**Background: **Out-of-hospital cardiac arrest (OHCA) is a life-threatening event. Thames Valley Air Ambulance supplement the statutory NHS ambulance service response, providing critical care by helicopter and critical care response vehicle. Interrogation of incidents for critical care dispatch is challenging. A timely response to OHCA is required to maximise life-saving opportunity. We hypothesised that by reducing call to at-patient time, the time saved would lead to favourable patient benefit and increased rate of critical care intervention.

**Aim: **To improve access to critical care by reducing call to at-patient time and attending a greater proportion of OHCA where critical care interventions are indicated.

**Method: **A pragmatic approach of ‘send and deescalate’ was proposed for critical care response vehicles for any incident where CPR instructions were attempted during the emergency call. The first trial was within a 30-min travel time, with a second trial within a 15-min travel time of the nearest critical care car. Cases were tagged as DOHCA30 and DOHCA15 for later identification. We monitored the number of OHCA, number of missed jobs, number of OHCA where airway and critical care interventions were undertaken, ROSC and stand-down rate. We also monitored the dispatch interval.

**Results: **There were 44 DOHCA30 taskings and 19 DOHCA15 taskings. On average, cases in the trial groups were dispatched one minute quicker (median 3-min, IQR 2–5 min) than cases outside of the trial (4-min, IQR 3–9.75 min), *p* = 0.003. There was no change in critical care intervention.

**Conclusion: **DOHCA30 and DHOCA15 did not increase the burden on wider service delivery. Whilst there was a clear benefit in reducing the dispatch interval, this was not clearly translated into delivery of critical care. Patient numbers were insufficient to determine any impact on ROSC. Exploration of demographics is warranted to understand factors that may influence dispatch of critical care assets.

## P56 Relationship between combat-related traumatic injury and 10-Year cardiovascular risk

### Usamah Haling^1^, Paul Cullinan^1^, Alexander N Bennett^2^, Nicola T Fear^3^, Anthony MJ Bull^4^, Christopher J Boos^5,*^ for the ADVANCE Study

#### ^1^National Heart and Lung Institute, Faculty of Medicine, Imperial College London, UK. ^2^Academic Department of Military Rehabilitation, Defence Medical Rehabilitation Centre, Stanford Hall Estate, Nottinghamshire, UK. ^3^The Academic Department of Military Mental Health, King’s College London, UK. ^4^Centre for Blast Injury Studies, Department of Bioengineering, Imperial College London, UK. ^5^Department of Cardiology, University Hospitals Dorset, Poole Hospital, Poole, UK

##### *Correspondence: christopher.boos@uhd.nhs.uk

***Scandinavian Journal of Trauma, Resuscitation and Emergency Medicine*** 2023, **31(1)**: P56

**Background: **Combat-related traumatic injury (CRTI) has been thought to negatively influence cardiovascular disease (CVD) trajectory. Its relationship to future predicted risk of CVD using a contemporaneous multi-marker CVD risk calculator has not been examined.

**Material and methods: **This was a baseline cross-sectional analysis of the Armed Services Trauma Rehabilitation Outcome (ADVANCE) Cohort Study^1,2^ in which adults with CRTI were compared to uninjured servicemen who were frequency-matched by age, sex, rank, deployment (Afghanistan 2003–2014) and role to the injured group. The comparative composite QRISK®3 predicted 10-year CVD risk (myocardial infarction or stroke) among the injured and uninjured groups were quantified on rested and fasted participants as part of their ADVANCE baseline visit.

**Results: 1134 **participants were recruited, which consisted of 578 injured and 556 uninjured men of similar age (mean 34.1 ± 5.4 years), ethnicity (90.6% Caucasian), time from deployment/injury (8.3 ± 2.1 years) and smoking history. Among the injured, blast (75.11%) was the commonest injury mechanism with a median NISS of 13.0 (IQR: 6–30). There were 161 limb amputees. Mental health illness (8.5% vs 4.3%; p = 0.004) and erectile dysfunction (11.6% vs 5.9%; *p* = 0.001) were more common in the injured versus uninjured respectively. Adjusted body mass index (28.1 ± 3.9 vs 27.4 ± 3.4 kg/m^2^; p = 0.001) and the blood pressure variance (2.1 [1.2–3.5] vs 1.7 [1.2–3.0] mmHg; *p* = 0.008) was also greater among the injured. There were no significant differences in the other QRISK®3 inclusion variables. The predicted 10-year CVD risk was similar between the injured (0.9% [0.4–1.8]) and uninjured (0.9% [0.4–1.7]) groups. The relative CVD risk (versus the age-matched population expected risk) was greater in both groups (1.6% [1.1–2.4] vs 1.5% [1.1–2.3]) respectively.

**Conclusion: **These findings suggest that CRTI is not associated with a relative increase in QRISK®3-calculated 10-year CVD risk. The longer-term CVD risk for this population remains unknown.

**Acknowledgments**

We wish to thank the research staff at both Headley Court and Stanford Hall who assisted with the ADVANCE study and, most importantly, the participants.

**Funding**

The ADVANCE study is funded through the ADVANCE Charity. Key contributors to this charity are the Headley Court Charity (principal funder), HM Treasury (LIBOR Grant), Help for Heroes, Nuffield Trust for the Forces of the Crown, Forces in Mind Trust, National Lottery Community Fund, Blesma—The Limbless Veterans and the UK Ministry of Defence**.**

**References**

1. ADVANCE Study: https://www.advancestudydmrc.org.uk/
2. Bennett AN, Dyball DM, Boos CJ, Fear NT, Schofield S, Bull AMJ, Cullinan P; ADVANCE Study. Study protocol for a prospective, longitudinal cohort study investigating the medical and psychosocial outcomes of UK combat casualties from the Afghanistan war: the ADVANCE Study. BMJ Open. 2020 Oct 30;10(10):e037850. doi:10.1136/bmjopen-2020-037850.

## P57 Management of blunt splenic injury: a five-year experience at a UK Major Trauma Centre

### Manasi Panshikar^1,*^, Zane Perkins^1^

#### ^1,*^Centre for Trauma Sciences, Queen Mary University of London, London, United Kingdom

##### *Correspondence: m.m.panshikar@smd17.qmul.ac.uk

***Scandinavian Journal of Trauma, Resuscitation and Emergency Medicine*** 2023, **31(1)**: P57

**Background: **Non-operative management (NOM) is the standard of care in haemodynamically stable patients with blunt splenic injuries (BSI), with selective use of adjunctive splenic artery embolisation (SAE) for high-grade BSI. However, considerable variation still exists in BSI management. This study provides a current picture of the management and outcomes of BSI patients treated at a single UK major trauma centre.

**Method: **This retrospective study separately reviewed all adult (> 16 years) and paediatric (≤ 16 years) patients with BSI admitted between October 2016 and October 2021. We report patient (survival) and treatment (treatment success/failure, complications) level outcomes for both cohorts.

**Results: **BSI was identified in 216 adult and 29 paediatric patients. In the adult cohort, 178 (82.4%) patients underwent NOM, of whom 69 (38.8%) were treated with adjunctive SAE, and 38 (17.6%) underwent splenectomy as the primary treatment. Of those who received NOM alone, 7/116 (6.0%) failed and 3/116 (2.6%) patients died. In the SAE group, 7/69 (10.1%) failed and 2/69 (2.9%) died. The only significant independent predictor of NOM failure in adults was the American Association for the Surgery of Trauma (AAST) grade of BSI severity. Adjunctive SAE only reduced NOM failure in patients with AAST grade V BSI. The incidence of complications following SAE was less than after splenectomy (25% vs 42%) and patients in the SAE group experienced significantly fewer systemic complications (17% vs 40%, *p* = 0.0172). In the paediatric cohort, 27 (93.1%) patients underwent NOM, of whom 2 (7.4%) received adjunctive SAE, and 2 (6.9%) were treated with emergency splenectomy. Primary treatment success and survival was 100% in the paediatric cohort.

**Conclusion: **Excellent NOM success and survival rates were observed in both adult and paediatric cohorts, however, superior outcomes were seen in paediatric patients. Adjunctive SAE only improved NOM success in patients with AAST grade V injuries.

## P58 Brain death and organ donation in post cardiac arrest patients treated with TTM in South Korea

### Soo Hyun Kim^1,*^, Semin Choi^2^, Young Shin Cho^3^

#### ^1,*^Department of Emergency Medicine, Eunpyeong St. Mary's hospital, Collage of Medicine, The Catholic University of Korea, Seoul, Korea. ^2^Department of Emergency Medicine, Uijeongbu St. Mary's Hospital, College of Medicine, The Catholic University of Korea, Uijeongbu-si, Korea. ^3^Department of Emergency Medicine, Soonchunhyang University Seoul Hospital, Seoul, Korea

##### *Correspondence: emksh77@gmail.com

***Scandinavian Journal of Trauma, Resuscitation and Emergency Medicine*** 2023, **31(1)**: P58

**Background: **The occurrence of brain death (BD) in patients with hypoxic-ischemic brain injury in post cardiac arrest patients creates opportunities for organ donation. We aimed to determine risk factors for evolution toward BD after OHCA.

**Method: **We analyzed all consecutive OHCA patients treated with TTM admitted to 12 medical centers at urban academic hospitals from October 2015 to December 2018. Survival and neurological outcomes (Glasgow–Pittsburgh Cerebral Performance Categories, CPC) were assessed at discharge. Good neurological outcome was defined as a CPC 1 or 2. BD was defined according to international guidelines. As recommended, BD was clinically diagnosed in the absence of confounding factors. Electroencephalogram was performed to confirm BD after complete TTM.

**Results: **A total 907 OHCA patients who were treated TTM were included in this study. Among these, 271 (29.9%) had good neurological outcomes, whereas 130 (14.3%) evolved toward BD. 61 (46.9%) of BD patients were performed organ donation. Independent risk factors for BD were age (OR 0.98 (0.97–1.00)), not-witnessed (OR 1.68 (1.05–2.67)), time from collapse to ROSC (OR 1.01 (1.00–1.02)), non-cardiac etiology (OR 2.76 (1.72–4.46)), no motor response (OR 2.48 (1.01–7.43)) and no PLR immediate after ROSC (OR 2.37 (1.19–5.19)). There were no differences in induction time, overcooling, cardiovascular score or 1st day fluid balance.

**Conclusion: **These findings might help physicians in their decision-making processes and could serve as a basis for developing a simple score that could accurately predict BD after OHCA treated with TTM. The early identification of a patient with BD might motivate teams to optimize organ protection in the prospect of organ donation rather than only considering withdrawal of life support.

## P59 The association between defibrillation using LIFEPAK vs ZOLL and survival following out-of-hospital cardiac arrest: a cohort study

### Carsten Meilandt^1,*^, Mette Qvortrup^2^, Bo Løfgren^3^, Morten T Bøtker^1^, Kasper G Lauridsen^3^

#### ^1^Prehospital Emergency Medical Services, Central Denmark Region, Aarhus, Denmark. ^2^Department of Cardiology, Viborg Regional Hospital, Denmark. ^3^Department of Medicine, Randers Regional Hospital, Denmark

##### *Correspondence: carmei@rm.dk

***Scandinavian Journal of Trauma, Resuscitation and Emergency Medicine*** 2023, **31(1)**: P59

**Background: **Defibrillation is essential to achieve return of spontaneous circulation (ROSC) following out-of-hospital cardiac arrest (OHCA) with shockable rhythms. Defibrillators commonly used in the Danish Emergency Medical Services (EMS) include the LIFEPAK 15 and the ZOLL X Series. These defibrillators use different biphasic waveforms and different maximal shock energies. This study aimed to investigate if the type of defibrillator used was associated with ROSC in OHCA.

**Methods: **This study included data on Danish adult OHCA patients subjected to at least one defibrillation by the EMS from 2016 to 2020. Data were extracted from the Danish Cardiac Arrest Registry and The National Patient Registry. Multivariable logistic regression adjusted for patient demographics (age, sex and comorbidities) and OHCA characteristics (initial rhythm, witnessed status, bystander cardiopulmonary resuscitation, use of AED prior to EMS arrival, EMS response time, location of arrest and prehospital physician involvement) was used to examine the association between defibrillator (LIFEPAK or ZOLL) and ROSC.

**Results: **Complete data were available for 5,485 of 6,498 patients. Overall, 1,310 (24%) were women, median (quartile 1; quartile 3) age was 70 (59; 79) and 3,353 (61%) had an initial shockable rhythm. ROSC was achieved in 3,119 (57%) patients. In total, 4,820 patients (88%) were defibrillated by the LIFEPAK, with 2,690 (56%) achieving ROSC while 665 patients (12%) were defibrillated by ZOLL, with 429 (65%) achieving ROSC. Patients defibrillated by ZOLL had an increased adjusted odds ratio (aOR) of ROSC compared to LIFEPAK (aOR 1.38; 95% CI: 1.15–1.65, *p* < 0.001). There was no significant difference in 30-day mortality (aOR 1.1; 95% CI: 0.9–1.3, *p* = 0.4).

**Conclusion: **Patients defibrillated by ZOLL X Series were more likely to achieve ROSC compared to those defibrillated by LIFEPAK 15 when adjusting for patient demographics and OHCA characteristics.

## P60 Association of socioeconomic deprivation with 30-day survival following out-of-hospital cardiac arrest in Scotland, 2011–2020

### Laura A.E. Bijman^1,*^; Rosemary C. Chamberlain^1^; Gareth Clegg^2^; Andrew Kent^3^; Nynke Halbesma^1^

#### ^1^Usher Institute, University of Edinburgh, Edinburgh, United Kingdom. ^2^Resuscitation Research Group, University of Edinburgh, Edinburgh, United Kingdom. ^3^Scottish Ambulance Service, Edinburgh, United Kingdom

##### *Correspondence: laura.bijman@ed.ac.uk

***Scandinavian Journal of Trauma, Resuscitation and Emergency Medicine*** 2023, **31(1)**: P60

**Background: **The pattern of higher incidence of out-of-hospital cardiac arrest (OHCA) in socioeconomically deprived areas is well-established, but patterning of subsequent survival is less clear. We quantified the crude and confounder adjusted association of socioeconomic deprivation with OHCA survival in Scotland.

**Method: **This was a population-based study of 20,913 non-traumatic, non-Emergency Medical Services (EMS) witnessed OHCAs with resuscitation attempted by the Scottish Ambulance Service (SAS), between April 2011 and March 2020. Deprivation was determined by Scottish Index of Multiple Deprivation (SIMD) of the home address of the patient. SIMD is an area based indicator of socioeconomic status based on the following seven domains; income, employment, health, education, access to services, crime and housing. For these analyses SIMD quintiles are used. Survival was measured at 30 days post-OHCA. Crude and confounder-adjusted associations of SIMD quintile with survival were estimated using logistic regression. Effect modification by age and sex was assessed by stratification.

**Results: **Crude analysis showed lower odds of 30-day survival in the most deprived quintile relative to least deprived (odds ratio (OR) 0.81, 95% confidence interval (CI) 0.69–0.96). Adjustment for age, sex and urban/rural residency decreased this OR to 0.63 (95%CI 0.53–0.75). A stronger association was observed in younger male age groups, and no association in males aged ≥ 80 years or any female age groups. With increasing deprivation, there were decreasing trends in the proportion of OHCAs with shockable initial cardiac rhythm and receiving bystander cardiopulmonary resuscitation (bCPR).

**Conclusion: **There is a socioeconomic disparity in OHCA survival in Scotland. This is not explained by confounding by age, sex or urban/rural residency. The deprivation-survival association is most pronounced in younger males. Initial cardiac rhythm and bCPR are potential mediators. It may be appropriate to target the most deprived areas with measures designed to increase bCPR to reduce this disparity in survival.

**Acknowledgements**

Author Laura A.E. Bijman is supported by a British Heart Foundation non-clinical PhD studentship (FS/19/74/34725). Author Nynke Halbesma is supported by a British Heart Foundation Intermediate Basic Science Research Fellowship (FS/16/36/32205). The Scottish OHCA Data Linkage Project is funded by the Scottish Government as part of the Scottish OHCA Strategy.

**Military abstracts**

## M1 Death on the urban battlefield: Preliminary analysis of 101 penetrating trauma deaths across the West Midlands (2012–2019)

### Nabeela S. Malik^1,2,*^, Douglas Bowley, Keith Porter, Emir Battaloglu

#### ^1^University Hospitals Birmingham NHS Trust, Birmingham, UK. ^2^212 (Yorkshire) Field Hospital, Sheffield, Yorkshire, UK. ^3^Royal Centre for Defence Medicine, Birmingham, UK

##### *Correspondence: nabeelamalik@nhs.net

***Scandinavian Journal of Trauma, Resuscitation and Emergency Medicine*** 2023, **31(1)**: M1

**Background: **Since 2014, a steep rise in violence-related penetrating trauma has been observed across the UK. Most published studies are hospital based, describing in-hospital mortality rates of 1.8–5%. However, homicide data report mortalities far in excess of these. This study aimed to describe the characteristics of patients who died from violence-related penetrating trauma in order to determine how mortality may be mitigated.

**Method: **In conjunction with HM Coroner and the Homicide Department, West Midlands Police, post mortem reports of adult and paediatric victims of violence-related penetrating trauma in the West Midlands (2012–2019) were obtained. Patient demographics, injury mechanism, weapon implicated and location of death were collated.

**Results: **101 patients were identified. 78.2% of victims were male, 22/101 (21.8%) were females. Median age of victims was 33 years (IQR 23.8–46.0), with 8.9% (9 victims) aged under 18. The majority of victims (89.1%) were injured due to a stabbing mechanism (one third of these were attributable to domestic knives) whilst 10.9% (n = 11) sustained gunshot wounds (pistol = 6, shotgun = 3). In half of all cases, causative weapon was unknown. Of the 101 deaths, two thirds (67/101) occurred in the pre-hospital setting, with 29 (28.7%) deaths occurring in the presence of a pre-hospital doctor. 24 patients (23.8%) had recognition of life extinct (ROLE) declared by paramedics. One third (33/101) of victims died in hospital, with 15 deaths (14.9%) occurring in ED and 18 (17.8%) during surgery.

**Conclusion: **Domestic knives appear to be the most commonly implicated weapon. Higher representation of females amongst homicide victims (21.8%) compared with hospital admissions (7%) may signifying higher case fatality amongst females, possibly relating to domestic violence. Two thirds of deaths occur in the pre-hospital setting and over 80% of deaths occur prior to encountering a surgeon. Further analysis of injury pattern, interventions received and potential preventability of death is underway.

## M2 A pilot study of construct validity of vessel exposure & haemorrhage control models

### Tim Stansfield^1,*^, Julian Scott^1^, Shervanthi Homer-Vanniasinkam^1^

#### ^1,*^Leeds Vascular Institute, Leeds, Yorkshire, UK

##### *Correspondence: tim.stansfield@nhs.net

***Scandinavian Journal of Trauma, Resuscitation and Emergency Medicine*** 2023, **31(1)**: M2

**Background: **Construct validity is an important criterion in effective skills assessment [1]. It measures the degree to which an assessed task can be used to infer the performer’s capability. In the case of surgical simulation, it measures how performance on the assessed simulator differentiates surgeons by level of experience. Vessel exposure and haemorrhage control is a technical skill included in the UK surgical curriculum [2] commenced at core speciality level. This pilot study aimed to determine the construct validity of two different haemorrhage control models (VI Box® and Medical Meats®) used in Yorkshire School of Surgery surgical training days.

**Method: **European Board of Vascular Surgery procedure specific and global rating scales were adapted for scoring performance on the haemorrhage control models. Surgical trainees were briefed on the models (inflow, outflow, targeting tube embedded in silicone, nature of injury, and the aim of the model: to demonstrate simulated vessel exposure and vessel repair). The trainee was then assessed using the model by a consultant surgeon. The assessor acted as an assistant without initiative. Time to completion, performance score within the procedural specific and global rating scale domains were recorded. Verbal feedback was then provided to the trainee.

**Results: **Four vascular trainees (2 different grades) were assessed on the VI box® haemorrhage control model. Separately, nine surgical trainees (2 different grades) were assessed on the Medical Meats® haemorrhage control model. There was a significant difference in the median performance scores on the procedure specific skills scale for trainees using the VI box® model (Mann–Whitney U test, *p* = 0.05).

**Discussion: **Construct validity is a viable criterion to assess in vessel exposure and haemorrhage control models. Future attempts to demonstrate construct validity will probably benefit from further model standardisation, a wider range of experience of those assessed and greater numbers of those assessed.

**References**

1. Norcini J, Anderson B, Bollela V, et al. Criteria for good assessment: consensus statement and recommendations from the Ottawa 2010 Conference. Med Teach. 2011; 33:206–14.
2. Intercollegiate Surgical Curriculum Programme: The new surgical curriculum for August 2021. Published online August 2021. https://www.iscp.ac.uk/iscp/curriculum-2021/. Accessed November 2022

## M3 Bluetooth tactical headsets improve the speed of accurate patient handoffs

### Daniel J. Stinner^1,*^, Cory McEvoy^2^, Michael A Broussard^3^, Aaron D Nikolaus^4^, Charles H. Parker^5^, Hector Santana^3^, Jason Karnopp^3^, Jigar A Patel^5^

#### ^1,*^Vanderbilt University Medical Center, Nashville, TN, USA. ^2^United States Army Special Operations Command, Fort Bragg, NC, USA. ^3^Joint Special Operations Command, Fort Bragg, NC, USA. ^4^Department of Anesthesiology, Naval Medical Readiness and Training Command, San Diego, CA, USA. ^5^Womack Army Medical Center, Fort Bragg, NC, USA

##### *Correspondence: Daniel.stinner@gmail.com

***Scandinavian Journal of Trauma, Resuscitation and Emergency Medicine*** 2023, **31(1)**: M3

**Background: **The Committee on En Route Combat Casualty Care recently ranked the patient handoff as their fourth research priority. Bluetooth technology has been introduced to the battlefield and has the potential to improve the tactical patient handoff. The purpose of this study is to compare the traditional methods of communication used in tactical medical evacuation by special operations medical personnel (radio push to talk (PTT) and Tactical Medic Intercom System (TM-ICS)) to Bluetooth communication.

**Methods: **Twenty-four simulated tactical patient handoffs were performed to compare Bluetooth and traditional methods of communication used in tactical medical evacuation. Patient scenario order and method of communication was randomized. Accuracy and time required to complete the patient handoff was determined. The study took place using a rotary wing aircraft kept at level 2 to simulate real-world background noise. Preferred method of communication for each study participant was determined.

**Results: **There was no difference in accuracy of the received patient handoffs between groups. There was also no difference in patient handoff transmission times at the ramp of the aircraft. However, when comparing patient handoff times to the medical team within the aircraft, Bluetooth voice activated transmission was significantly faster than both TM-ICS and Radio PTT, while Bluetooth PTT and Radio PTT were also significantly faster than TM-ICS. Bluetooth communication was also ranked as the preferred method of patient handoff in all study participants.

**Conclusion: **This study demonstrates that Bluetooth headsets used by medical providers during a simulated patient handoff to a special operations medical team resulted in faster patient handoffs without sacrificing accuracy, thus allowing for faster time to initiation of further medical treatment.

## M4 Biomechanical validation of the field-expedient pelvic splint

### Jonathan C. Savakus^1^, Thomas Skacel^2^, Michael Jindia^2^, Yazan Al-Madani^2^, Liam Spoletini^2^, Daniel J Stinner^1, *^

#### ^1^Department of Orthopaedic Surgery, Vanderbilt University Medical Center. ^2^Department of Biomedical Engineering, Vanderbilt University

*Correspondence: Daniel.stinner@gmail.com. ***Scandinavian Journal of Trauma, Resuscitation and Emergency Medicine*** 2023, **31(1)**: M4

**Background: **Pelvic ring injuries can be acutely life threatening. A technique to improvise an effective pelvic binder in an austere environment, known as a Field-Expedient Pelvic Splint (FEPS), has been described [1]. This technique utilizes supplies routinely carried by medics – the Combat Application Tourniquet (C-A-T®) and the SAM® Splint. We hypothesize that the FEPS will be biomechanically equivalent to a commercially available pelvic binder.

**Method: **Force generation potential of the FEPS was measured utilizing a commercial load frame (Instru-Met Corporation). A commercially obtained *SAM Pelvic Sling* was utilized as a control group for initial force generation. The FEPS was tested in triplicate for three separate metrics: initial force generation, persistence of force generation over a 6 h longitudinal test period, and initial force generation after repeated assembly/disassembly.

**Results: **The FEPS was capable of generating 203N (± 7N) with one windlass crank and 420N (± 34N) with two windlass cranks. The *SAM Pelvic Sling* generated 197N (± 11N) of initial force. There was no statistically significant difference between FEPS after one windlass crank and *SAM Pelvic Sling*, but the force generated by the FEPS with two windlass cranks was significantly higher than the *SAM Pelvic Sling.* Longitudinal testing showed that initial force generation of the FEPS was 338N (± 20) at time zero, and by hour 6 the force generated had decreased to 189N (± 19N)*.* Reusability testing showed no significant difference with force generation by the FEPS after repeated assembly/disassembly with one crank of the windlass.

**Conclusion: **The FEPS is capable of exerting similar pelvic compressive forces to its commercial equivalent. This force generation persists at effective levels over a 6-h time course. The FEPS is a viable alternative in the austere or resource-limited environment for temporarily stabilizing a pelvic fracture.

**Reference**

1. Savakus J, Gehring A, Stinner DJ. Field-expedient pelvic splint: a technique for the resource-limited environment. BMJ Mil Health. 2021 Jun 14. Online ahead of print.

